# Pharmacological PIK3C2B inhibition rescues XLMTM phenotype in mouse models and identifies molecular markers of disease

**DOI:** 10.1172/jci.insight.198568

**Published:** 2026-04-09

**Authors:** Andrew Shearer, Melissa L. Brooks, Maxine M. Chen, Thiwanka Samarakoon, John Hsieh, Gramoz Kondakci, Emanuele Perola, Jason Brubaker, Kristina Fetalvero, Stefanie Schalm, Joana Caetano-Lopes

**Affiliations:** 1 Blueprint Medicines Corporation, Cambridge, Massachusetts, USA.; 2 Bayer Research & Innovation Center, Cambridge, Massachusetts, USA.

**Keywords:** Development, Genetics, Muscle biology, Drug therapy, Neuromuscular disease, Protein kinases

## Abstract

X-linked myotubular myopathy (XLMTM) is a rare genetic disorder that typically presents at birth with progressive muscle weakness and respiratory difficulties and is caused by myotubularin 1 (*MTM1*) gene mutations. Here, we examine the role of phosphatidylinositol-4-phosphate 3-kinase catalytic subunit type 2-β (PIK3C2B), a lipid kinase that interacts with MTM1, in XLMTM in various models. We examined the effect of BLU3797, a highly potent, selective, orally bioavailable PIK3C2B inhibitor, on survival, muscle development, myofiber phenotypes, and gene expression in *MTM1^–/y^* mice. PIK3C2B-deficient XLMTM animals demonstrated increased survival, restored muscle function, fewer myofibers with centralized nuclei, and normalization of disease-associated molecular markers. BLU3797 alleviated the XLMTM phenotype in a dose-dependent and reversible manner. Loss of functional PIK3C2B in XLMTM mice promoted a more differentiated, adult-like myofiber profile, which was strongly associated with normalization of disease surrogates and a reduction in markers of early muscle development and regeneration. BLU3797 treatment appears to modulate the expression of miRNAs associated with satellite cell activation and myofiber fusion. These findings indicate that PIK3C2B inhibition with BLU3797 effectively reverses the XLMTM disease phenotype by enhancing muscle function and promoting development toward a more mature state.

## Abstract 2

Inhibition of lipid kinase PIK3C2B by novel compound BLU3797 restores muscle function and increases survival in animal models of X-linked myotubular myopathy.

## Introduction

X-linked myotubular myopathy (XLMTM) is a rare genetic disorder with an estimated prevalence of 1 in 50,000 male births and is characterized by profound muscle weakness and respiratory challenges (1). XLMTM primarily affects males, with symptoms typically appearing at birth or in early infancy. Affected individuals exhibit profound muscle weakness, leading to difficulties with movement and motor skills. Hypotonia, or low muscle tone, is a common feature, resulting in floppiness and poor muscle control (2–6). Respiratory complications, such as breathing difficulties and frequent respiratory infections, are also prevalent in patients with XLMTM (4–8). Additionally, patients may experience hepatobiliary disease, feeding difficulties, delayed or loss of motor milestones, and skeletal abnormalities, among other issues (4–6, 8–10). Supportive therapies, including respiratory support, physical therapy, and nutritional interventions, play a crucial role in symptom management and promoting overall well-being. These interventions offer much-needed progress in addressing the challenges associated with XLMTM and improving the lives of those affected by the condition (4, 5, 8, 11). Despite advances in disease intervention and supportive care, there is currently no cure for XLMTM, warranting the need to develop curative therapies.

The underlying cause of XLMTM is deleterious mutations in the myotubularin 1 (*MTM1*) gene that result in loss of function or altered expression of myotubularin, a lipid phosphatase. To date, over 300 mutations in the *MTM1* gene have been identified (2, 12–18). Although information on genotype-phenotype relationships is limited, truncating mutations typically result in a more severe phenotype, whereas nontruncating mutations outside the catalytic domain of myotubularin have been associated with milder presentations (2, 12, 14, 15, 19–21). Myotubularin is a broadly expressed phosphatase that plays a critical but cryptic role in muscle development and function (16). The essential function of myotubularin and its orthologs in regulating phosphatidylinositol 3-phosphate (PtdIns3P), endocytosis, and endo(lyso)somal function has been extensively studied in various model organisms, including *Drosophila*, *C*. *elegans*, zebrafish, mice, and higher mammals (16). These models have provided valuable insights into the role of myotubularin and have contributed to our understanding of myotubularin deficiency (22). The major enzymatic function of myotubularin is to dephosphorylate PtdIns3P and phosphatidylinositol 3,5-bisphosphate, which are important phosphoinositides involved in membrane trafficking and signaling (20, 23–25). Mutations in the *MTM1* gene lead to a deficiency or dysfunction of myotubularin, disrupting normal muscle cell maturation and function (26). Deficiency of myotubularin has also been linked to elevated mTORC1 activity, leading to a disruption in the connection between starvation and the initiation of autophagy (27).

Several physiological changes are observed in the muscle tissues of patients with MTM1 deficiency and in animal models. The *MTM1*-KO mouse differs from the human disease phenotype by exhibiting muscle weakness and atrophy primarily during the postnatal period, implying that myotubularin deficiency may primarily affect muscle growth and maintenance rather than muscle development in these mice (22). The *MTM1*p.R69C mouse model was generated by introduction of a point mutation (c.205C>T) to exon 4 of *MTM1*, resulting in a loss of *MTM1* full-length mRNA expression, and animals exhibited early muscle atrophy and muscle weakness at 2 months of age. Similar to the KO model, histopathological analysis revealed small myofibers with centrally located nuclei, which is a characteristic feature observed in the human disease (28). The exact mechanisms by which *MTM1* mutations cause XLMTM are still being elucidated, but it is believed that impaired membrane trafficking, abnormal muscle fiber formation, and altered signaling pathways contribute to the disease phenotype (23, 26, 29, 30).

A reciprocal interplay has been demonstrated between MTM1 and the PI3K enzyme family (20, 31). Phosphatidylinositol-4-phosphate 3-kinase catalytic subunit type 2-β (PIK3C2B) is a ubiquitously expressed member of the PI3K family that plays a critical role in intracellular signaling pathways. PIK3C2B catalyzes the phosphorylation of phosphatidylinositol (PtdIns) and phosphatidylinositol-4-phosphate (PtdIns4P), generating PtdIns3P and PtdIns(3,4)P2, which serve as important signaling molecules within the cell (32). The relationship between PIK3C2B and MTM1 was initially established through in vivo experiments, where mice deficient for both MTM1 and PIK3C2B had no observable disease phenotype (31, 33). A recent study indicates that MTM1 and PIK3C2B both metabolize a shared pool of PtdIns3P within endosomal compartments. In the absence of MTM1, PtdIns3P accumulates on vesicles positive for early endosome antigen 1, while depletion of PIK3C2B helps restore PtdIns3P levels (34). These observations highlight the pivotal role of MTM1 in regulating a distinct pool of PtdIns3P.

Here, we present additional in vitro and in vivo data that further support the role of PIK3C2B inhibition in reversing XLMTM symptoms in a mouse model and describe the downstream impacts of PIK3C2B inhibition on gene expression alterations that may affect development of the disease phenotype.

## Results

### BLU3797 is a selective and potent PIK3C2B inhibitor.

An internal discovery program targeting inhibition of PIK3C2B led to the synthesis of inhibitors with various degrees of potency and selectivity. Two of these inhibitors, BLU3797 and BLU2720, were extensively characterized in a series of in vitro and in vivo studies.

**Figure 1:**
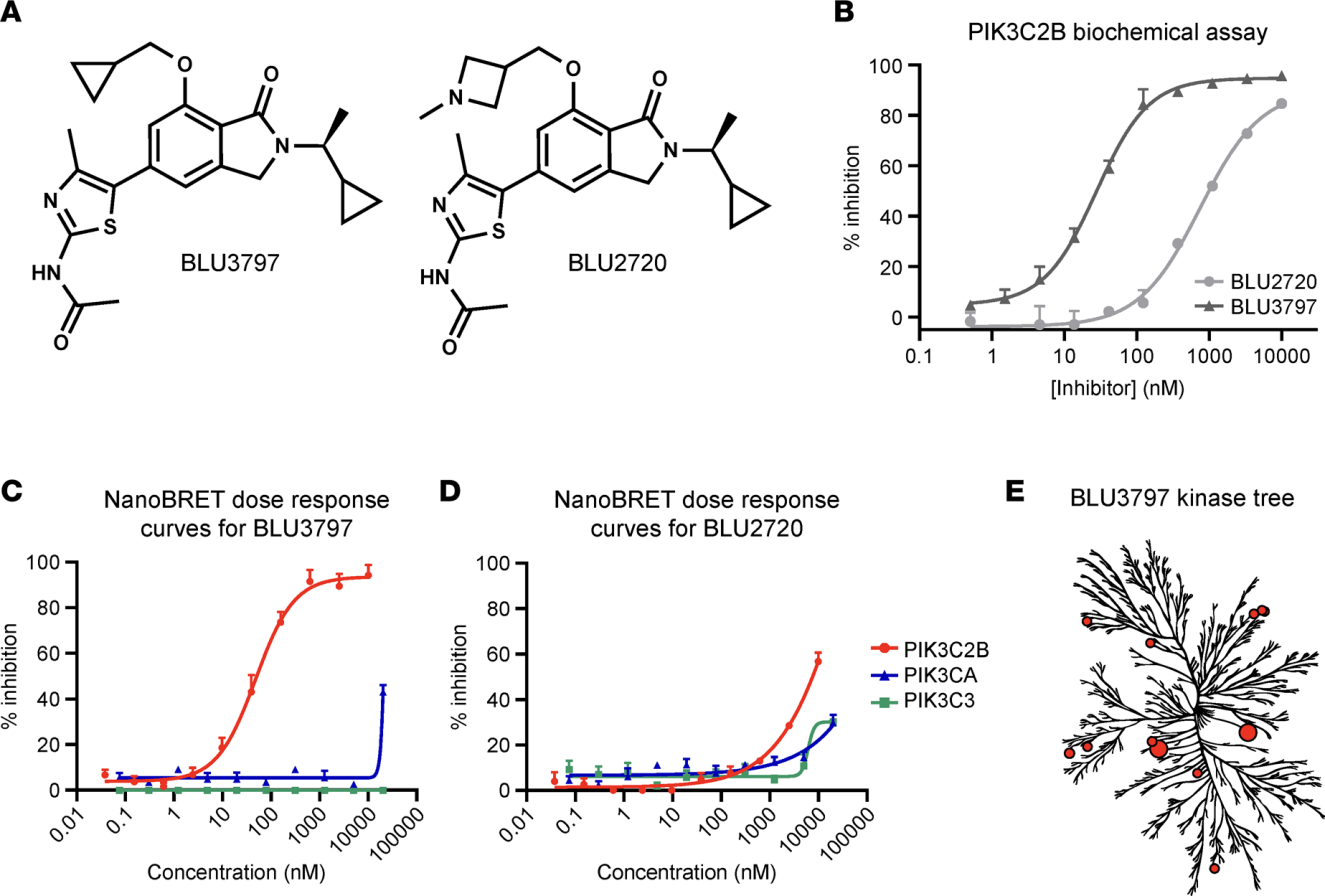
BLU3797 is a selective and potent inhibitor of human PIK3C2B kinase. (**A**) Chemical structures of BLU3797 and BLU2720. (**B**) Biochemical analysis of PIK3C2B inhibitory activity of BLU3797 (IC_50_ = 28 nM) and BLU2720 (IC_50_ = 1.0 μM) in the presence of 1 mM ATP. (**C** and **D**) NanoBRET-based determination of BLU3797 and BLU2720 PI3K engagement. Individual points are mean ± SEM. (**E**) Kinome tree representing the reactivity of BLU3797 to other kinases as assessed by KINOMEscan (Eurofins). Red dots represent kinases with significant BLU3797 interaction; dot size is proportional affinity. The S(10) of BLU3797 is 0.015, indicating high selectivity.

BLU3797 showed high selectivity for PIK3C2B while sparing other PI3K family members, along with pharmacokinetic properties that would furnish in vivo exposures in mice achieving C_trough_ = PIK3C2B IC_50_ over a 24-hour period utilizing a single orally administered dose. BLU2720 possesses physicochemical properties and selectivity similar to BLU3797 as assessed biochemically (Figure 1B). Further evaluation of BLU3797 (Figure 1C) and BLU2720 (Figure 1D) by NanoBRET revealed levels of selectivity similar to those observed biochemically (Table 1). BLU3797 had low affinity for other kinases in general as assessed by kinome scan (Figure 1E), with an overall S(10) selectivity score of 0.015. Taken together, these data imply that BLU3797 is a potent and highly kinome-sparing inhibitor of PIK3C2B.

### BLU3797 treatment increases MTM1-null myotube nuclear density.

C2C12 cells are a common cell model to evaluate muscle development, and when engineered to lack MTM1 expression, previous publications (35) have shown that they have reduced myogenic potential. Our own generation of a series of *MTM1*-null clones showed heterogeneity in terms of myogenic identity even as myoblasts (Supplemental Figure 1; supplemental material available online with this article; https://doi.org/10.1172/jci.insight.198568DS1). Control clones that had been transfected with nontargeting sgRNA showed signs of lost myogenic identity (low Pax7 and myogenin protein levels) and showed limited fusion (data not shown) when differentiated.

**Figure 2:**
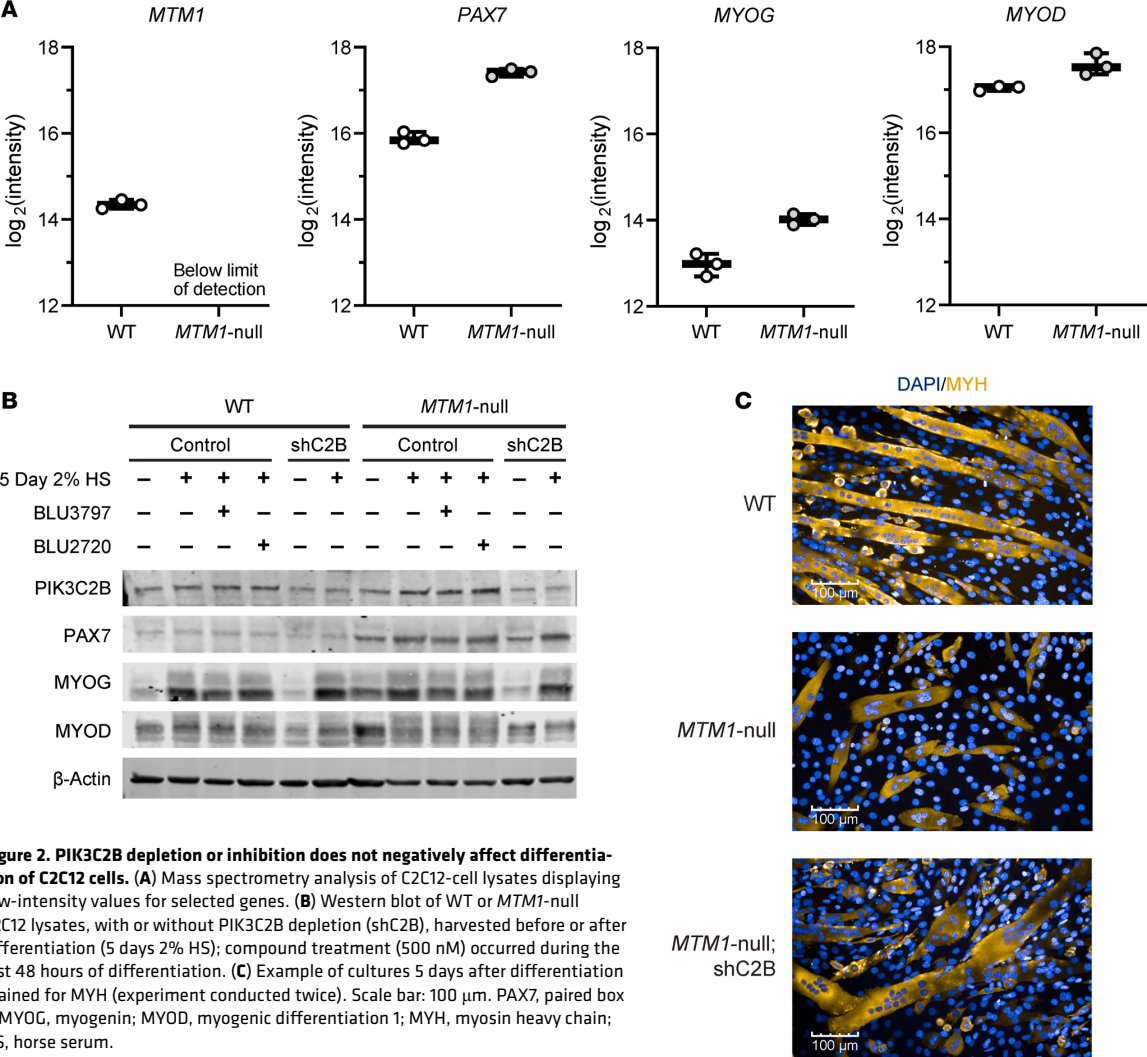
PIK3C2B depletion or inhibition does not negatively affect differentiation of C2C12 cells. (**A**) Mass spectrometry analysis of C2C12-cell lysates displaying raw-intensity values for selected genes. (**B**) Western blot of WT or *MTM1*-null C2C12 lysates, with or without PIK3C2B depletion (shC2B), harvested before or after differentiation (5 days 2% HS); compound treatment (500 nM) occurred during the last 48 hours of differentiation. (**C**) Example of cultures 5 days after differentiation stained for MYH (experiment conducted twice). Scale bar: 100 μm. PAX7, paired box 7; MYOG, myogenin; MYOD, myogenic differentiation 1; MYH, myosin heavy chain; HS, horse serum.

**Figure 3:**
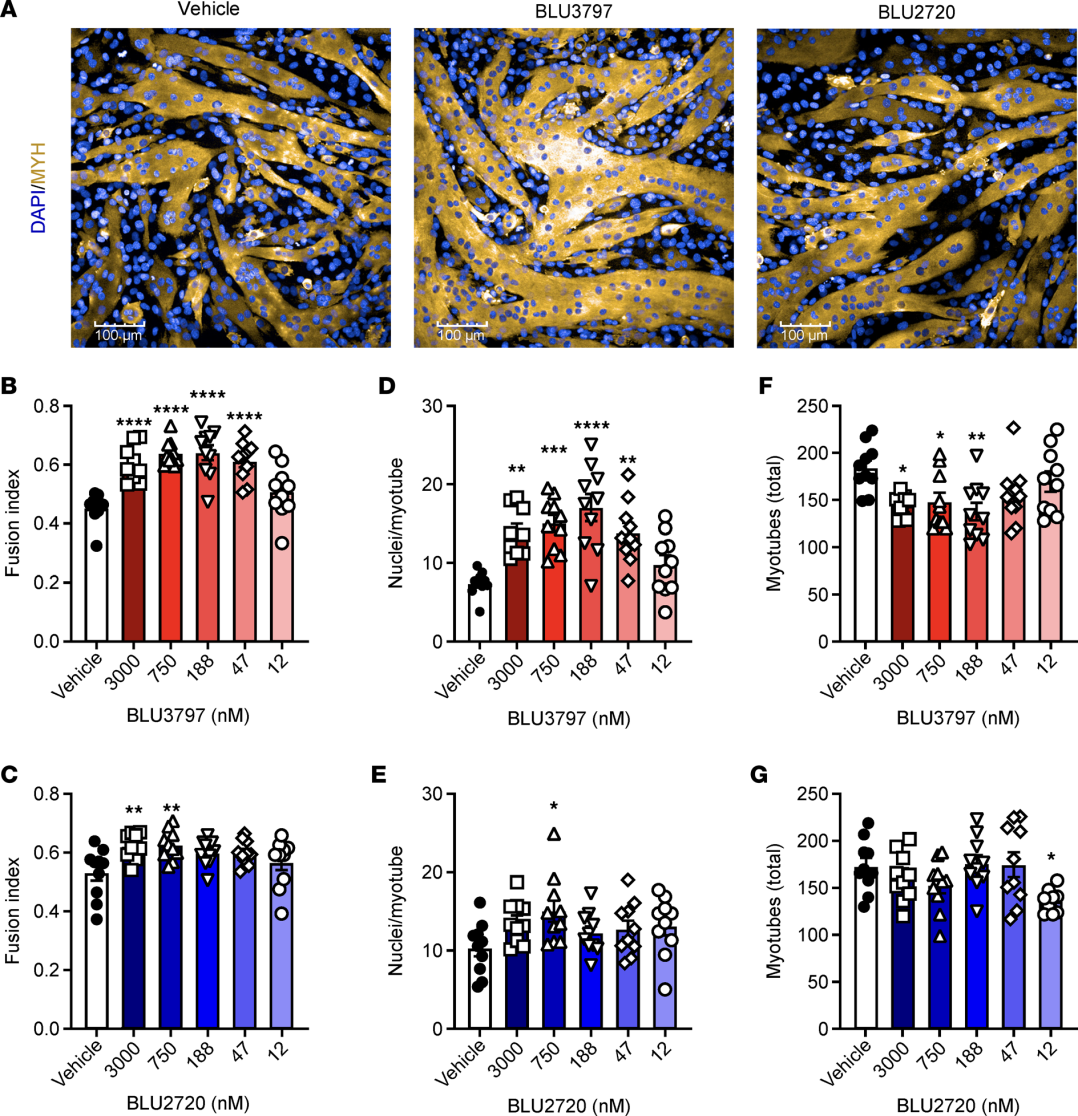
PIK3C2B inhibition promotes myocyte-myotube fusion in C2C12 cells. (**A**) Example images of *MTM1*-null C2C12 cells fixed after 5 days of differentiation (2% HS) and 3 μM of BLU3797 or BLU2720 treatment for the final 48 hours (experiment conducted 4 times). Scale bar: 100 μm. (**B** and **C**) Representative quantification (3 independent experiments) of the fraction of nuclei in MYH-positive structures (fusion index) from 5 random nonoverlapping fields (*n* = 10). (**D** and **E**) Representative quantification (3 independent experiments) of average nuclei in MYH-positive structures (myotubes) from 5 random nonoverlapping fields (*n* = 10). (**F** and **G**) Representative quantification of the total MYH-positive structures (myotubes) from 5 random nonoverlapping fields (*n* = 10). Data shown as mean ± SEM. *P ≤ 0.05, **P ≤ 0.01, ***P ≤ 0.001, ****P ≤ 0.0001. Comparisons were done with 1-way ANOVA with Dunnett’s correction for multiple comparisons. MYH, myosin heavy chain; HS, horse serum.

A single clone exhibiting both acceptable lineage commitment and fusion competence was selected for further analysis. Mass spectrometry of undifferentiated C2C12 cells confirmed that MTM1 was present in WT cells but absent in the *MTM1*-null cells (Figure 2A). In *MTM1*-null cells, there was a marked increase in PAX7 expression and more moderate increases in myogenin (MYOG) and myogenic differentiation, findings that were corroborated by Western blot analysis (Figure 2B). Upon differentiation, MYOG levels increased in both WT and *MTM1*-null cells, and treatment with BLU3797 or BLU2720 did not affect these levels. WT and *MTM1*-null cells transduced with shRNA targeting *Pik3c2b* resulted in a 50% reduction in PIK3C2B expression. After 5 days of differentiation, *MTM1*-null myotubes appeared wider compared with WT cells. Additionally, and in line with previously published data (36), *MTM1*-null cells showed reduced terminal differentiation, as indicated by an apparent lower number of multinucleated myotubes, a phenotype that was improved by *Pik3c2b* shRNA (Figure 2C). We then used *MTM1*-null cells to create an assay that would evaluate whether there was any effect upon fusion or terminal differentiation with a PIK3C2B inhibitor (Figure 3A). Treatment with BLU3797 increased the fusion index, the number of nuclei within sarcomeric myosin-positive objects, in a dose-dependent fashion (Figure 3B). Although there appeared to be an increase in this metric with BLU2720 as well, the change from baseline was not as significant, reflecting the difference in potency of PIK3C2B inhibition between the 2 molecules (Figure 3C). There was also a significant dose-dependent increase in the average number of nuclei per myotube (Figure 3, D and E). Overall, the greatest increase in myotube nuclei was observed at the 188 nM concentration, suggesting that higher doses did not provide additional benefit. In contrast to BLU3797, treatment with BLU2720 did not result in a substantial increase in nuclei per myotube. Intriguingly, the total number of myotubes in the BLU3797-treated conditions decreased in a dose-dependent manner (Figure 3, F and G). This reduction was inversely related to the increase in myonuclear number, a pattern not observed with BLU2720. As expected, these trends were absent in *MTM1*-null cells with *Pik3c2b* knockdown (Supplemental Figure 2), suggesting that the observed effects are specifically associated with PIK3C2B inhibition.

### PIK3C2B loss of function rescues the disease phenotype in the MTM1^–/–^ mouse model.

**Figure 4:**
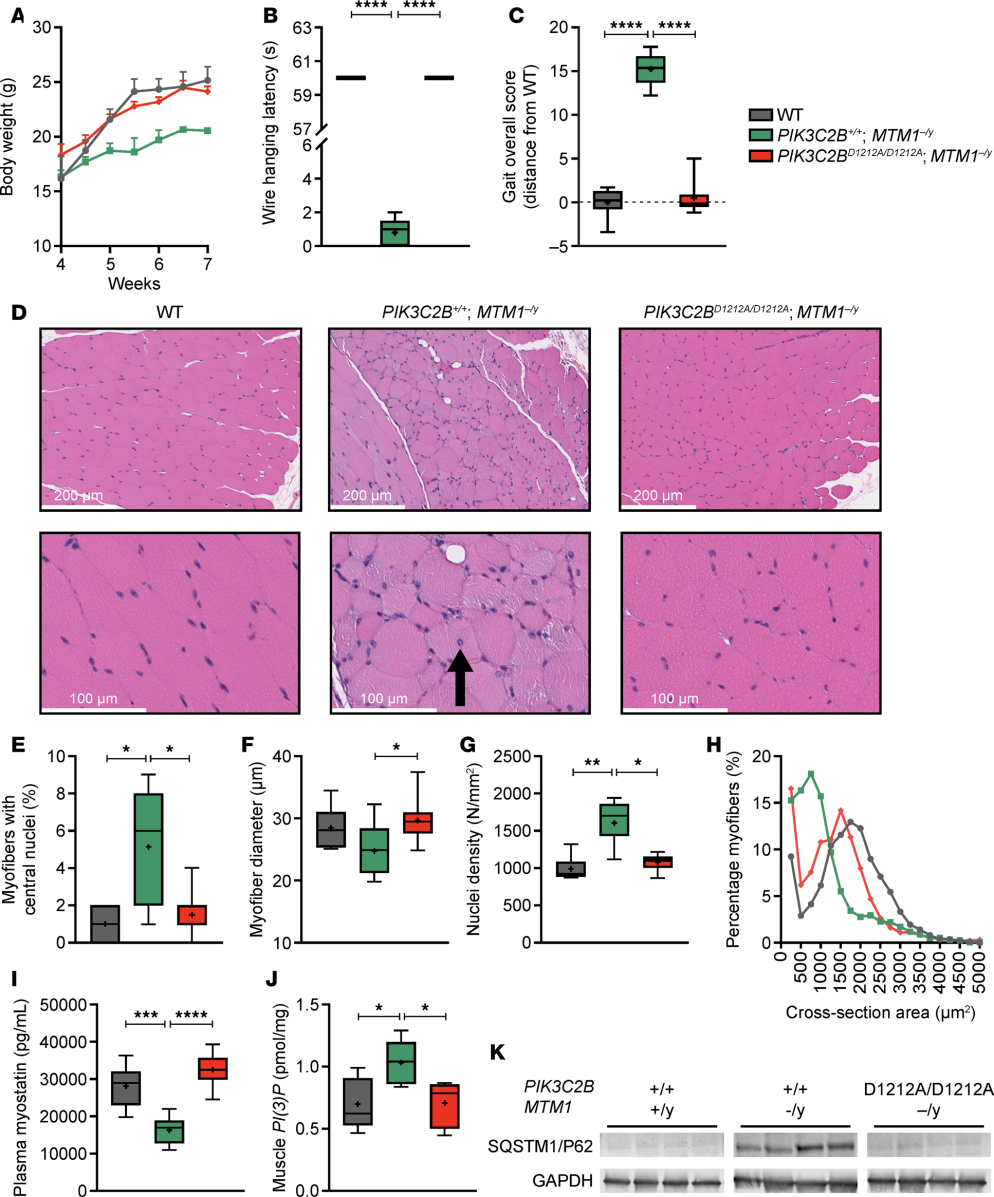
In a mouse model of XLMTM (*MTM1^–/y^*), the presence of the PIK3C2B kinase-dead mutation leads to the restoration of muscle function and histology. (**A**) Comparison of male *MTM1^–/y^*, WT, and PIK3C2B kinase-dead (PIK3C2B^D1212A/D1212A^) *MTM1^–/y^* weight gain. (**B**) Analysis of muscle strength by wire hanging latency. (**C**) Comparison of mobility and muscle coordination by kinematic gait analysis. (**D**) Representative images of H&E-stained gastrocnemius muscle (experiment conducted 3 times). Arrow indicates myofiber with central nucleus. Scale bars: 100 μm and 200 μm. (**E**) Summary of histological analysis comparing percentage of myofibers with centralized nuclei. (**F**) Summary of histological analysis examining myofiber diameter. (**G**) Summary of histological analysis of nuclear density within a tissue section. (**H**) Histogram profile of the cross-sectional area of myofibers. (**I**) Plasma myostatin level comparison. (**J**) Muscle PtdIns3P level comparison. (**K**) Western blot staining of SQSTM1/P62 from muscle lysates. Data shown as mean ± SEM. In box-and-whisker plots, median (line), mean (symbol), 25th–75th quartile range (box), and minimum and maximum (whiskers) are shown. Number of animals per group ranged from 5 (*PIK3C2B^+/+^MTM1^–/y^*) to 10 (WT and *PIK3C2B^–/–^MTM1^–/y^*). *P < 0.05; **P < 0.01; ***P < 0.001; ****P < 0.0001. Comparisons of wire hanging latency, percentage of myofibers with central nuclei, myofiber diameter, plasma myostatin, and myofiber nuclei density were done with Kruskal-Wallis test with Dunn’s correction for multiple comparisons. Comparisons of kinematic gait analyses were done with 1-way ANOVA with Tukey’s correction for multiple comparisons. Comparisons of myofiber diameter and muscle PtdIns3P expression were done with 1-way ANOVA with Holm-Šídák’s correction for multiple comparisons. PtdIns3P, phosphatidylinositol 3-phosphate.

We generated a kinase-dead (D1212A) PIK3C2B-expressing mouse to assess the effect of global PIK3C2B activity reduction (Supplemental Figure 3A). As expected, introduction of the *D1212A* mutation (Supplemental Figure 3B) did not affect PIK3C2B protein expression (Supplemental Figure 3, C–D) and had no impact on mouse survival, gross phenotype, weight (Supplemental Figure 3E), or fertility. This mouse strain was then bred with the *MTM1^–/y^* mouse line to better understand the extent of rescue that would be expected from treatment with a PIK3C2B-selective inhibitor. As reported by other groups (31, 33), there was a profound rescue of the disease phenotype with loss of kinase activity. Specifically, loss of functional PIK3C2B in the context of XLMTM led to the restoration of normal growth (Figure 4A) and muscle function as assessed by wire hanging (Figure 4B). Additionally, we observed that the gait of XLMTM animals lacking functional PIK3C2B was restored to resemble that of WT controls (Figure 4C).

In addition to the improvements in growth and muscle function, there was also a near-complete improvement in the histological phenotypes observed in XLMTM (Figure 4D). The percentage of myofibers with centralized nuclei, a common marker of XLMTM, was elevated in mice deficient for MTM1, and this elevation was fully reduced to WT levels in animals that were also deficient for PIK3C2B activity (Figure 4E). A similar effect was also observed for myofibers, where the diameter was decreased in MTM1-deficient mice compared with WT controls but was increased back to that of WT controls by the lack of PIK3C2B activity (Figure 4F). Because the myofiber diameter was reduced in *MTM1^–/y^* mice, there was also an observed increase in the number of nuclei per unit of area, which was fully corrected by the loss of functional PIK3C2B (Figure 4G). The overall cross-sectional area of myofibers was also revealed to be significantly decreased in *MTM1^–/y^* mice relative to WT, and this phenotype was largely corrected by loss of functional PIK3C2B (Figure 4H). Both the histological and functional data indicated that loss of functional PIK3C2B corrected the XLMTM-associated muscle phenotype. We then investigated changes in molecular markers of disease to further understand this effect.

Previous work has demonstrated that levels of myostatin, an inhibitor of muscle growth, are decreased in both the plasma and muscle tissue of animal models of centronuclear myopathies, including XLMTM (37). Indeed, *MTM1^–/y^* mice showed a decrease in plasma myostatin levels that was restored to WT levels with loss of PIK3C2B activity (Figure 4I). The marker PtdIns3P, which is known to be elevated in *MTM1*-null muscle, was fully reduced by loss of PIK3C2B activity (Figure 4J). An additional marker commonly seen in *MTM1^–/y^* is the accumulation of SQSTM1/P62 owing to stalled autophagy. SQSTM1/p62 is a key selective autophagy receptor that recognizes and binds ubiquitinated protein aggregates and damaged organelles. Its levels also serve as an indicator of autophagic flux, with accumulation reflecting impaired autophagy (38). Indeed, SQSTM1/P62 protein levels were significantly increased in *MTM1*-null animals but reduced to WT levels with the loss of functional PIK3C2B (Figure 4K).

### Loss of PIK3C2B in MTM1^–/y^ animals led to notable alterations in metabolic myogenic proteins.

**Figure 5:**
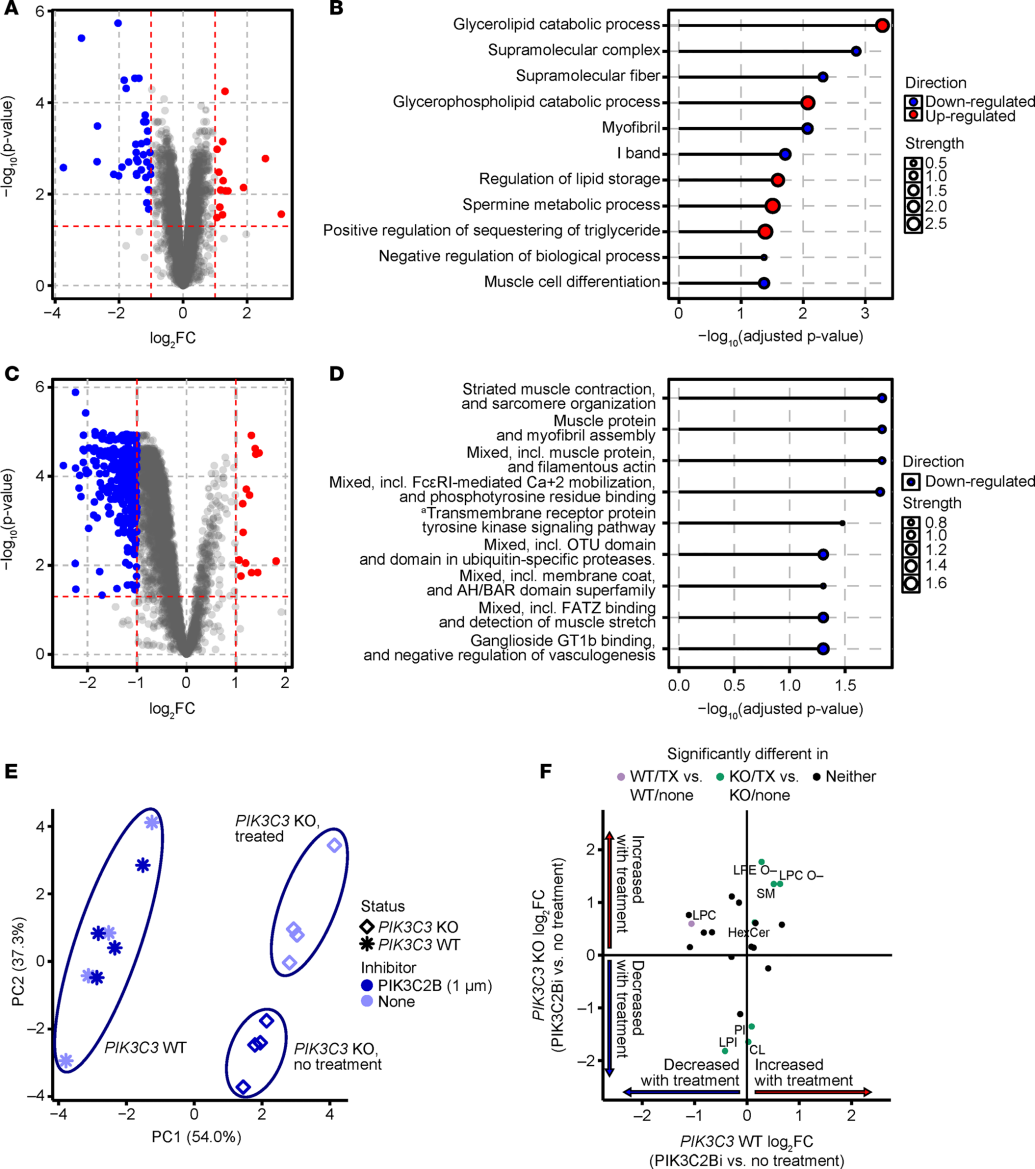
Proteomics and phosphoproteomics reveal decreases in abundance of muscle-related proteins in PIK3C2B kinase-dead *MTM1-*KO mice compared with *PIK3C2B* intact *MTM1*-KO mice. (**A**) Differential proteome abundance in *MTM1^–/y^/PIK3C2B^D1212A/D1212A^* mice (*n* = 6) compared with *MTM1^–/y^/PIK3C2B^+/+^* mice (*n* = 5). (**B**) Significantly enriched (adjusted *P* < 0.05) GO pathways in the proteome of *MTM^–/y^/PIK3C2B^D1212A/D1212A^* mice. (**C**) Differential phosphoproteome abundance in *MTM1^–/y^/PIK3C2B^D1212A/D1212A^* mice compared with *MTM1^–/y^/PIK3C2B^+/+^* mice. (**D**) Significantly enriched (adjusted *P* < 0.05) GO pathways in the phosphoproteome of *MTM ^–/y^ /PIK3C2B^D1212A/D1212A^* mice. ^a^Transmembrane receptor protein tyrosine kinase signaling pathway, including FcεRI-mediated Ca^2+^ mobilization. (**E**) Principal component analysis of *PIK3C3*-KO and *PIK3C3*-WT C2C12 cells that were treated with PIK3C2B inhibitor (1 μM) or not treated (*n* = 4/group). (**F**) Congruence between fold-change differences in *PIK3C3*-KO cells treated with BLU3797 (1 μM) versus *PIK3C3*-KO cells without treatment and *PIK3C3*-WT cells treated with PIK3C2B inhibitor (1 μM) versus *PIK3C3*-WT cells without treatment. Comparison of lipid abundance profiles was done with Bayes moderated 2-tailed *t* tests. Significance assessed at *P* < 0.05.

Proteomic and phosphoproteomic analyses using mass spectrometry were performed to uncover additional changes and identify potential disease-relevant markers (Supplemental Figure 4). Both in vitro and in vivo findings suggested that there was an increase in myogenesis or, at least, an accumulation of larger myofiber or myotubular structures after the loss of PIK3C2B activity. Unexpectedly, loss of PIK3C2B activity in *MTM1^–/y^* animals led to a noticeable decrease in myogenic markers (Figure 5, A and B), accompanied by an increase in proteins associated with particular types of metabolism. This pattern has also been reported by others (39, 40) and is generally used to distinguish developing or regenerating muscle tissue. In line with these findings, we observed decreased phosphorylation (Figure 5, C and D) of contractile proteins and calcium-mobilizing factors. Similar changes have also been documented in XLMTM diseases models (41) and are thought to reflect maladaptive remodeling of contractile structures. Collectively, these results suggest that loss of PIK3C2B activity in XLMTM muscle promotes normal tissue maturation without affecting lineage commitment.

Increased PtdIns3P levels have been used to generally describe the underlying mechanism behind the development of the XLMTM disease phenotype. Additional studies, largely in vitro, have found the nature of PIK3C2B-related PtdIns3P levels to be highly contextual and spatially restricted within the cell (31, 34). Phosphatidylinositol 3-kinase catalytic subunit type 3 (PIK3C3), or VPS34, is a lipid kinase that is involved not only in the synthesis of PtdIns3P but also in endomembrane dynamics, but unlike PIK3C2B, the loss of PIK3C3 results in loss of embryonic viability (31). We performed lipidomic profiling of WT and *PIK3C3^–/–^* C2C12 cells with and without PIK3C2B compound treatment and identified 21 lipids in these cell lines, including phospholipids (*n* = 14), sphingolipids (*n* = 3), glycerolipids (*n* = 2), and sterolipids (*n* = 2). Treatment of WT C2C12 cells with BLU3797 had minimal impact on overall lipid composition, whereas loss of PIK3C3 resulted in a pronounced alteration of the cellular lipid profile (Figure 5E). The addition of BLU3797 further modified the lipid profile, resulting in an intermediate phenotype that clustered between WT and *PIK3C3*-KO cells without treatment. Comparison of the differential abundance profiles between BLU3797 treatment and no treatment in *PIK3C3*-KO cells versus *PIK3C3*-WT cells showed that, unlike the lack of significant treatment effect in the *PIK3C3*-WT cells, a PIK3C2B inhibitor significantly changed (7/21, 33%) the lipid groups profiled compared with no treatment in *PIK3C3*-KO cells (Figure 5F).

### Selective inhibition of PIK3C2B effectively rescues the MTM1-null phenotype.

*MTM1^–/y^* mice were backcrossed with C57BL/6 WT mice over several generations, resulting in a genetic background that was 70% C57BL/6 as determined by SNP analysis. This shift led to a marked increase in disease severity and a decrease in animal survival compared with previously published studies (42). Animals began to succumb to disease around the time of weaning (P21), with most requiring humane euthanasia within 2–3 days afterward. This period of mouse development is characterized by marked muscle growth, including myofiber lengthening, physiological hypertrophy, and fiber type maturation occurring nearly simultaneously (43–45). This shift in background created a more pronounced form of the disease, similar to what has been observed in other muscle-related diseases (42, 46). We generated a conditional version of this model, which did not result in loss of viability or the XLMTM disease phenotype. However, it did show a reduction in myofiber diameter and an increase in the fraction of myofibers with centralized nuclei (Supplemental Figure 5).

**Figure 6:**
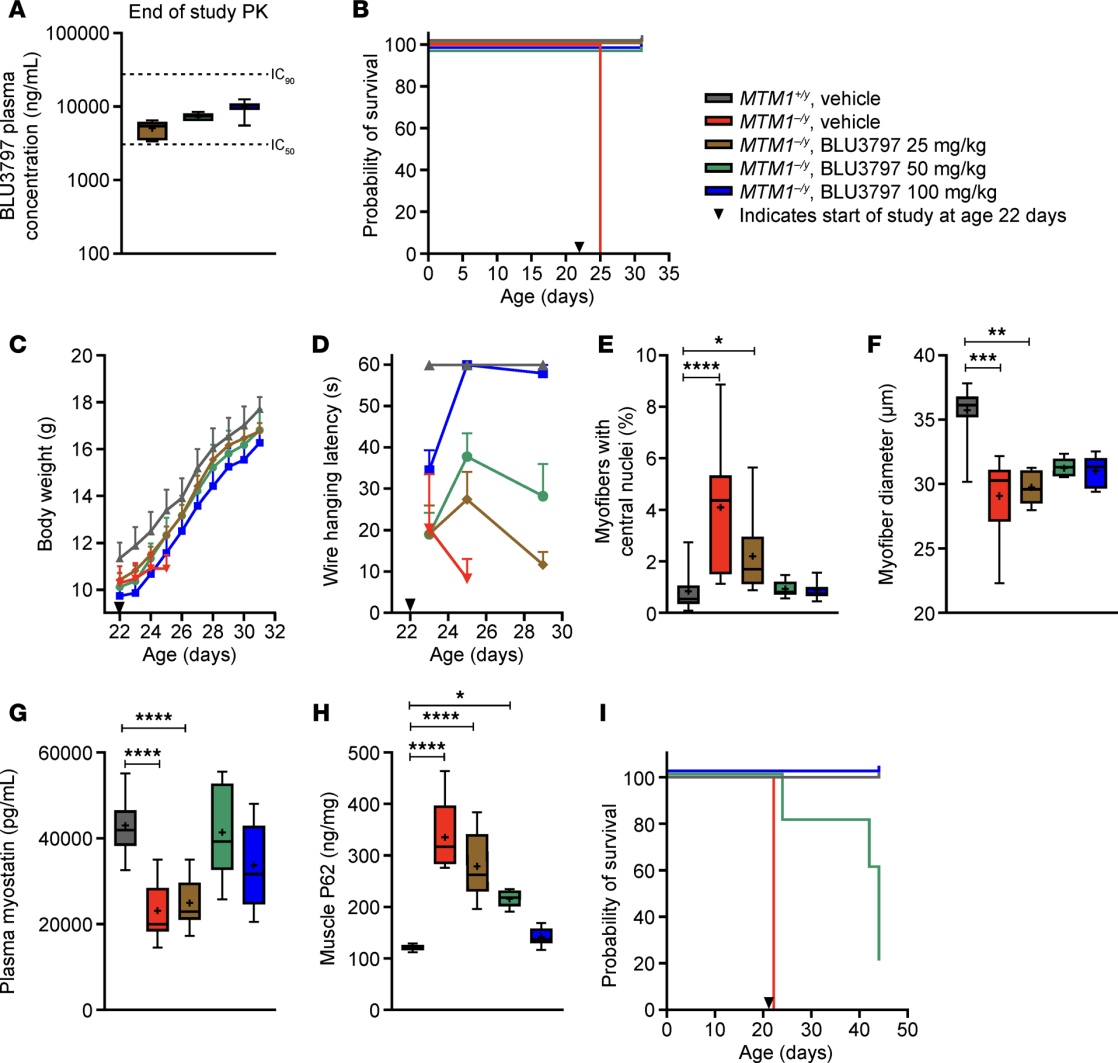
BLU3797 extends the lifespan of *MTM1^–/y^* male mice, reinstates muscle function, and improves histological characteristics. (**A**) A 10-day study of plasma PK of BLU3797 administered orally once daily at 22 days of age. FBS-corrected NanoBRET IC_50_ and IC_90_ values are indicated. (**B**) Kaplan–Meier survival curve. *MTM1^–/y^* vehicle control group reached humane endpoints 3 days after the study start date. (**C**) Body weight. (**D**) Assessment of muscle strength by wire hanging latency. (**E**) Summary of histological analysis of muscle samples examining the percentage of myofibers with centralized nuclei and the (**F**) myofiber diameter. (**G**) Plasma myostatin levels and (**H**) muscle SQSTM1/P62 levels (both detected by ELISA). (**I**) Kaplan–Meier survival curve for an extended 28-day study, where BLU3797 was dosed once every 48 hours. *MTM1^–/y^* vehicle control group reached humane endpoints 3 days after the study start date. Arrowheads represent start of the study. Data shown as mean ± SEM. In box-and-whisker plots, median (line), mean (symbol), 25th–75th quartile range (box), and minimum and maximum (whiskers) are shown. The number of animals per group ranged from 5 to 7. *P < 0.05; **P < 0.01; ***P < 0.001; ****P < 0.0001. Comparisons of wire hanging latency, percentage of myofibers with central nuclei, myofiber diameter, plasma myostatin, and myofiber nuclei density were made with a Kruskal-Wallis test with Dunn’s correction for multiple comparisons. PK, pharmacokinetics.

Given the constraints associated with this model’s survival, we adapted our in vivo studies to begin at P22. Increasing daily doses of BLU3797 (25, 50, 100 mg/kg) all seemed to surpass the NanoBRET-calculated IC_50_ (Figure 6A) and led to a marked improvement in animal survival through the end of the study (Figure 6B). Animals treated with this compound gained weight and developed in a fashion similar to WT controls, whereas untreated *MTM1^–/y^* animals showed a failure to thrive, as evidenced by their inability to gain weight, requiring humane euthanasia (Figure 6C). All treated groups showed improvement in wire hanging latency, with the degree and durability of this effect showing a strong dose dependency (Figure 6D). Analysis of tissue sections also found a dose-dependent decrease in the number of myofibers with centralized nuclei to the level of WT tissue (Figure 6E). Further analysis revealed a modest increase, but not a full rescue, in myofiber diameter (Figure 6F). Compound treatment normalized the level of plasma myostatin (Figure 6G) and the level of muscle SQSTM1/P62, as detected by ELISA (Figure 6H). We further investigated whether daily dosing was needed to rescue the XLMTM phenotype by dosing animals once every 48 hours at 50 or 100 mg/kg. As expected, both groups showed a significant improvement in survival (Figure 6I), which was persistent in the higher-dose group but began to wane in the 50 mg/kg group. By the end of the study, *MTM1^–/y^* animals treated with 100 mg/kg of BLU3797 had a 100% survival rate, while those treated with 50 mg/kg had a 20% survival rate.

We next considered whether the durability and extent of rescue were not only dose dependent, but also influenced by the timing of treatment initiation. In a small-scale study, animals started on treatment either just after weaning (22 days of age) or at the typical time of euthanasia (25 days of age), receiving twice-daily doses of 75 mg/kg BLU3797 to achieve C_trough_ at the NanoBRET-derived IC_90_. This regimen resulted in rescued viability and improved muscle function (Supplemental Figure 6, A–D). After treatment, animals were withdrawn from the compound and monitored until humane euthanasia was required. The decline in muscle function after the final dose was highly similar between the 2 groups. Myostatin levels in the D25 group were lower than those in the D22 group, yet remained improved compared with the vehicle-treated group (Supplemental Figure 6E). Curiously, even after compound withdrawal, SQSTM1/P62 levels remained significantly reduced at the end of the study (Supplemental Figure 6F). This finding indicates that similar results are achieved whether the animals are dosed preventively or therapeutically.

### miRNA analysis of tissues.

**Figure 7:**
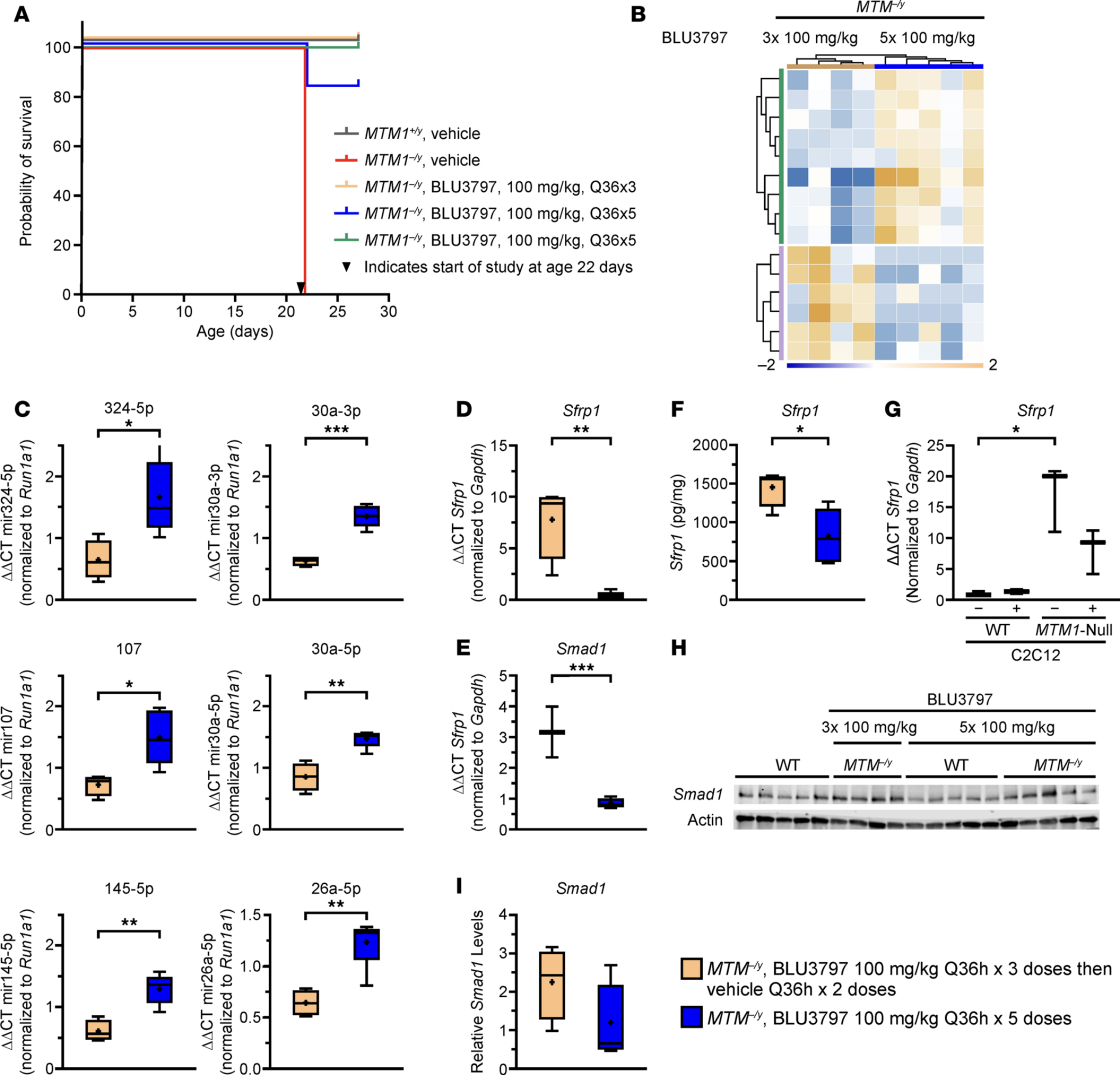
PIK3C2B inhibition alters miRNA expression and decreases muscle SFRP1 protein levels. (**A**) Survival of *MTM1^–/y^* mice treated with BLU3797 at the indicated number of times following a once every 36 hours dosing schedule. Arrowhead represents start of the study. (**B**) Clustering of treatment groups by differential miRNA expression. (**C**) Validation of select miRNAs by quantitative real-time PCR (qRT-PCR). All sample values are normalized to *Rnu1a1* expression and relative expression in WT mice treated with BLU3797. (**D** and **E**) qRT-PCR analysis of *Sfrp1* and *Smad1* levels normalized to *Gapdh* and expression in WT mice treated with BLU3797. (**F**) SFRP1 protein levels from *MTM1^–/y^* mouse muscle treated with and without BLU3797. (**G**) qRT-PCR of *Sfrp1* expression in WT C2C12 cells or deficient for MTM1 with BLU3797 treatment (final 48 hours of 5-day protocol); values normalized to *Gapdh* and WT untreated cells. (**H**) Western blot of SMAD1 and total actin in lysates from the muscle of *MTM1^–/y^* mice. (**I**) Quantification of SMAD1 levels shown in **H**; values are normalized to actin and protein levels in WT mice treated with BLU3797. In box-and-whisker plots, median (line), mean (symbol), 25th–75th quartile range (box), and minimum and maximum (whiskers) are shown. *P ≤ 0.05, **P ≤ 0.01, ***P ≤ 0.001. Comparisons of select miRNAs across BLU3797 dosing regimens were done with an unpaired 2-tailed *t* test. Comparisons of SFRP1 protein levels were made using Dunnett’s correction.

Our data suggests that there is a critical window during muscle development when PIK3C2B inhibition is effective and fully reversible. Identifying dynamic markers of disease has been challenging, and those currently used are somewhat tangential to the disease phenotype (e.g., SQSTM1/P62). miRNAs have been put forward as potential markers to assess disease severity. Therefore, we examined how the muscle miRNA profile changes under different treatment conditions in the context of the disease phenotype. To accomplish this, we treated WT and XLMTM animals with BLU3797 every 36 hours, starting at day 22 of age, administering either 5 or 3 doses (Figure 7A). Comparisons between WT and XLMTM were complicated by the large number of miRNAs that showed differential expression. Comparing the miRNA profiles of animals that received continuous treatment (5 doses) with those that underwent withdrawal (3 doses) revealed a smaller set (15) of differentially expressed miRNAs (Figure 7B). Of the 15 identified miRNAs, 9 (miRNAs 103, 107, 324-5p, 145, 30a, 1198, 125a-3p, 26a, and 490) were increased in the 5-dose group compared with the 3-dose group. The remaining 6 miRNAs were decreased in the 5-dose group relative to the 3-dose group (miRNAs 802, M55-1, 292-5p, 511 2133, and 1186b). We were particularly interested in 6 miRNAs whose expression levels in the 5-dose group matched those of the WT group but were differentially modulated in the 3-dose group (miRNAs 103, 107, 324-5p, 145, 30a, and 26a). We then focused on those miRNAs for which commercially available quantitative PCR (qPCR) kits were available (Figure 7C). Of the selected miRNAs, 3 have human homologs known to be involved in muscle development and maturation (30a-3p, 30a-5p, and 26a-5p) (47–49), while the other 3 appeared to have a less clearly defined role in muscle development (324-5p, 107, and 145-3p) (50, 51). All 6 miRNAs were found to be increased in the 5-dose group relative to the 3-dose group, confirming our initial screening results.

To gain deeper mechanistic insight into how the loss of miRNA expression may contribute to the disease phenotype, we focused on the expression of secreted frizzled-related protein 1 (SFRP1) and SMAD1. SFRP1 has been shown to inhibit myotube formation in both C2C12 cell cultures and primary muscle tissue and is believed to be regulated by miR-30a-3p (52–54). SMAD proteins are known to play a role in muscle development, and the overexpression of SMAD1 has been shown to inhibit muscle cell differentiation (55). Consistent with the reduction in suppressive miRNAs, transcript levels of both genes were significantly elevated in the XLMTM withdrawal group but decreased with continuous BLU3797 treatment (Figure 7, D and E). SFRP1 protein levels in muscle, assessed by ELISA, were reduced in the continuous dosing group relative to the withdrawal group (Figure 7F). Building on these findings, we observed that *Sfrp1* transcript levels were increased in our *MTM1*-null C2C12 cells, and this increase was partially corrected with BLU3797 treatment (Figure 7G). *Smad1* mRNA, but not SMAD1 protein, levels were significantly modulated by continuous treatment (Figure 7, H and I). Collectively, these data suggest that the identified miRNAs are part of a broader regulatory mechanism. When dysregulated in XLMTM, this leads to increased expression of factors that can affect terminal differentiation of myoblasts, a process that can be corrected through PIK3C2B inhibition.

## Discussion

In this study, we developed a potent and selective PIK3C2B inhibitor that phenocopied the loss of functional PIK3C2B by dramatically rescuing the XLMTM phenotype in mice. In both models, XLMTM animals with deficient PIK3C2B activity exhibited increased survival, restored muscle function, fewer myofibers with centralized nuclei, and normalized disease-associated molecular markers (SQSTM1/P62 and myostatin). The improvements observed with BLU3797 treatment were dose dependent and reversible, supporting the conclusion that inhibition of PIK3C2B kinase activity can effectively rescue the XLMTM phenotype.

We found that *MTM1*-null C2C12 cells, regardless of PIK3C2B depletion, exhibited similar levels of lineage commitment. However, *MTM1*-null cells with PIK3C2B depletion showed an increased number of nuclei per myotube. Consistent with this, treatment with BLU3797 significantly increased the number of nuclei per myotube, accompanied by a modest but significant decrease in the number of unique myotubes. This suggests that enhanced myotube-myotube and terminal myoblast-myotube fusion contribute to this effect. It is therefore not surprising that the critical period when PIK3C2B activity is most detrimental is when its inhibition is most disease modifying.

The precise role of PIK3C2B in XLMTM disease progression is linked to excessive PtdIns3P production, though this effect may be limited to specific spatial or contextual settings. Supporting this finding, overall relative proportions of phospholipids in C2C12 cells remained unchanged after PIK3C2B inhibition unless PIK3C3 was depleted. A recent study demonstrated that MTM1 is essential for regulating phosphoinositide metabolism in C2C12 cells, with a notable influence on PtdIns3P distribution and turnover within endosomal compartments (34) and, similar to our own findings, terminal differentiation. Depleting PIK3C2B in these cells restored PtdIns3P levels, corrected the trafficking defects, and increased terminal differentiation. Collectively, these findings suggest that lack of functional MTM1 triggers a cascade of events that may disrupt the ability of activated satellite cell progeny (myoblasts) to fuse with existing myofibers. This hypothesis aligns with the developmental timing of XLMTM manifestation in both humans and experimental models, which coincides with a phase of rapid postnatal growth dependent on satellite cells and potentially tertiary myoblast populations (44, 56).

To identify potential early or dynamic markers of XLMTM, we compared the miRNA profiles of muscle tissue from animals treated with, and subsequently withdrawn from, BLU3797. This analysis revealed a distinct set of miRNAs whose expression levels changed in the disease state and normalized after treatment. We observed increased *Srfp1* and *Smad1* transcript levels in XLMTM muscle, which correlated with reduced levels of mir30a-3p/5p and mir26a-5p. Inhibition of PIK3C2B increased mir30p/5p and mir26a-5p levels and ultimately decreased expression of SMAD1 and SFRP1 proteins. Notably, these changes occurred alongside an opposite trend in myostatin levels, suggesting that the regulation of these factors may become uncoupled in XLMTM, potentially contributing to disease pathology.

Loss or inhibition of functional PIK3C2B is closely associated with normalization of disease markers and reduction in indicators of early muscle development or regeneration. To target this pathway, we developed BLU3797, a selective kinase inhibitor that specifically blocks PIK3C2B while sparing other lipid kinases. Using the *MTM1^–/y^* mouse model of human XLMTM, treatment with BLU3797 produced a dose-dependent improvement of disease symptoms, including rescue in survival and muscle function. Our data further show that altering dosing parameters directly influenced these outcomes. By testing BLU3797 as both a therapeutic intervention and a preventative of disease onset in mice, we demonstrated that our treatment window is broad and adaptable to a heterogenous disease and patient population. A limitation of our study is in the use of the wire hanging test to evaluate muscle function. Given the complex behavior associated with wire hanging, and other factors that may influence test results (e.g., weight and balance), it is not always possible to relate the outcome of the test to a sole neuromuscular defect.

In summary, our findings demonstrate that selective inhibition of PIK3C2B, using compounds such as BLU3797, can normalize disease biomarkers, improve muscle function, and enhance survival in preclinical models of XLMTM. These results provide compelling evidence that targeting PIK3C2B may address key aspects of disease pathology, including the restoration of myoblast fusion and muscle regeneration. However, although the *MTM1^–/y^* mouse model offers valuable insights into the molecular mechanisms underlying XLMTM, it does not fully capture the complexity and heterogeneity of the disease as it manifests in humans. Therefore, further research in larger mammals, such as canine models and ultimately humans, is essential to validate the therapeutic potential of PIK3C2B inhibition. Continued investigation will be critical for advancing PIK3C2B inhibitors toward clinical application and for realizing their potential as part of the therapeutic strategy for XLMTM.

## Methods

### Sex as a biological variable

XLMTM is an X-linked genetic disorder, with the *MTM1* gene being present on the X chromosome and primarily affecting males. Phenotypes were compared between male and female WT and KO animals, and as expected, differences were observed between male and female animals. Experiments were therefore conducted in male animals with WT versus *MTM1^–/y^* genotypes.

### Generation of PIK3C2B small molecule inhibitors

Key steps in the synthesis of (S)-N-(5-(2-(1-cyclopropylethyl)-7-(cyclopropylmethoxy)-1-oxoisoindolin-5-yl)-4-methylthiazol-2-yl) acetamide (BLU3797; Figure 1A) are shown in Supplemental Figure 7A.

#### Step 1: Synthesis of (S)-5-bromo-2-(1-cyclopropylethyl)-7-(cyclopropylmethoxy)isoindolin-1-one.

To a solution of (S)-5-bromo-2-(1-cyclopropylethyl)-7-fluoroisoindolin-1-one (Intermediate 3, 100 mg, 0.335 mmol) and cyclopropylmethanol (121 mg, 1.68 mmol) in N,N-dimethylformamide (DMF; 1 mL), sodium tert-pentoxide (55.4 mg, 0.503 mmol) was added at 25°C, and the mixture was stirred at 110°C for 1 hour. The reaction mixture was concentrated under reduced pressure and the residue purified by prep-TLC (petroleum ether/ethyl acetate) to furnish the titled compound as a white solid (100 mg, 85%). For liquid chromatography–mass spectrometry (LCMS), *m/z* = 350 [M+H]^+^.

#### Step 2: Synthesis of (S)-N-(5-(2-(1-cyclopropylethyl)-7-(cyclopropylmethoxy)-1-oxoisoindolin-5-yl)-4-methylthiazol-2-yl) acetamide.

To a mixture of (S)-5-bromo-2-(1-cyclopropylethyl)-7-(cyclopropylmethoxy)isoindolin-1-one (step 1, 40 mg, 0.114 mmol) and N-(4-methylthiazol-2-yl)acetamide (17.8 mg, 0.114 mmol) in DMF (2 mL) was added Pd(OAc)_2_ (2.56 mg, 0.011 mmol) and Cs_2_CO_3_ (74.4 mg, 0.228 mmol) and tri-tert-butylphosphonium tetrafluoroborate (6.63 mg, 0.023 mmol) at 25°C, and the reaction mixture was stirred at 100°C for 12 hours under N_2_. The reaction mixture was concentrated under reduced pressure and the residue was purified by *prep*-HPLC to furnish the titled compound as a brown solid (21.7 mg, 38%). LCMS: *m/z* = 426 [M+H]^+^; ^1^H NMR (400 MHz, DMSO-d_6_) δ: 7.22 (s, 1H), 7.03 (s, 1H), 4.65–4.52 (m, 2H), 4.05 (d, 2H), 3.72–3.61 (m, 1H), 2.44 (s, 3H), 2.24 (s, 3H), 1.39 (d, 4H), 1.20–1.10 (m, 1H), 0.72–0.62 (m, 3H), 0.51 (tt, 1H), 0.47–0.39 (m, 3H), 0.38–0.32 (m, 1H).

Key steps in the synthesis of (S)-N-(5-(2-(1-cyclopropylethyl)-7-((1-methylazetidin-3-yl)methoxy)-1-oxoisoindolin-5-yl)-4-methylthiazol-2-yl)acetamide (BLU2720; Figure 1A) are shown in Supplemental Figure 7B.

#### Step 1: 5-bromo-2-[(1S)-1-cyclopropylethyl]-7-[(1-methylazetidin-3-yl)methoxy]isoindolin-1-one.

To a solution of 5-bromo-2-[(1S)-1-cyclopropylethyl]-7-fluoro-isoindolin-1-one (100 mg, 335.40 μmol, 1 *eq*) and (1-methylazetidin-3-yl)methanol (169.62 mg, 1.68 mmol, 5 *eq*) in DMF (1 mL) was added sodium 2-methylbutan-2-olate (55.41 mg, 503.10 μmol, 1.5 *eq*). The mixture was stirred at 110°C for 1 hour under N_2_. LCMS showed desired MS. The reaction mixture was concentrated under reduced pressure and purified by *prep*-HPLC to afford 5-bromo-2-[(1S)-1-cyclopropylethyl]-7-[(1-methylazetidin-3-yl)methoxy]isoindolin-1-one as a yellow oil (110 mg, 290.02 μmol, 86.47% yield). LCMS: *m/z* = 379.1 [M+H]^+^.

#### Step 2: N-[5-[2-[(1S)-1-cyclopropylethyl]-7-[(1-methylazetidin-3-yl)methoxy]-1-oxo-isoindolin-5-yl]-4-methyl-thiazol-2-yl]acetamide.

To a solution of 5-bromo-2-[(1S)-1-cyclopropylethyl]-7-[(1-methylazetidin-3-yl)methoxy]isoindolin-1-one (30 mg, 79.10 μmol, 1 *eq*) and N-(4-methylthiazol-2-yl)acetamide (13.59 mg, 87.00 μmol, 1.1 *eq*) in DMF (1 mL) was added Pd(OAc)_2_ (1.78 mg, 7.91 μmol, 0.1 *eq*), Cs_2_CO_3_ (51.54 mg, 158.19 μmol, 2 *eq*), and tri-tert-butylphosphonium tetrafluoroborate (2.29 mg, 7.91 μmol, 0.1 *eq*). The mixture was stirred at 100°C for 12 hours under N_2_. The reaction mixture was concentrated under reduced pressure and purified by *prep*-HPLC to furnish N-[5-[2-[(1S)-1-cyclopropylethyl]-7-[(1-methylazetidin-3-yl)methoxy]-1-oxo-isoindolin-5-yl]-4-methyl-thiazol-2-yl]acetamide as a yellow solid (8.2 mg, 15.32 μmol, 19.36% yield, 93.5% purity). LCMS: *m/z* = 455.1 [M+H]^+1^; H-NMR (400 MHz, CD_3_OD) *δ* ppm 8.55 (s, 1H), 7.31 (s, 1H), 7.08 (s, 1H), 4.73–4.55 (m, 2H), 4.38 (br s, 2H), 4.28–4.05 (m, 4H), 3.73–3.58 (m, 1H), 3.09 (br s, 1H), 2.85 (br s, 3H), 2.44 (s, 3H), 2.33–2.17 (m, 3H), 1.45–1.23 (m, 4H), 1.22–1.11 (m, 1H), 0.76–0.62 (m, 1H), 0.56–0.45 (m, 1H), 0.44–0.36 (m, 1H), 0.32 (td, 1H, *J* = 4.4, 9.2 Hz).

### PIK3C2B biochemical assay

The potency of compounds against the in vitro enzymatic activity of human PIK3C2B was determined by ADP-Glo format (Promega). The lipid kinase reaction was performed in 10 mM MgCl_2_, 100 mM NaCl, 1 mM EGTA, 0.03% CHAPS, 2 mM DTT, and 50 mM HEPES, at pH 7.5. To initiate the enzyme reaction, 120 nM of human PIK3C2B enzyme (PV3574l, Thermo Fisher Scientific) and 2 mM ATP (Sigma-Aldrich) were preincubated with compounds at 2× final concentration for 10 minutes, followed by the addition of an equal volume of 300 nM lipid substrate phosphatidylinositol diC8 (P-0008, Echelon Biosciences) to a final concentration of 60 nM PIK3C2B, 1 mM ATP, and 150 nM diC8. ADP generated during the kinase reaction was resynthesized to ATP, which was determined as a luminescence signal in a coupled luciferase/luciferin reaction with an EnVision Multimode Plate reader (Revvity). Data were normalized to background control (0.5% DMSO) and 100% inhibition controls (10 μM BGT-226), and the IC_50_ was calculated using a 4-parameter logistic equation.

### Kinome selectivity score determination

The kinase selectivity of BLU3797 was determined at 3 μM using the KINOMEscan screening platform (Eurofins). The S(10) score is the ratio calculated from the number of nonmutant kinases bound by the test compound over the total number of nonmutant kinases at the testing concentration, where 10% residual activity is used as the threshold.

### Target engagement assay

The NanoBRET Target Engagement assay was utilized to determine target engagement in live cells. Target protein was expressed as a fusion with NanoLuc luciferase, and the ability of a compound of interest to displace a fluorescent tracer was performed following the manufacturer’s (Promega) instructions.

In this manuscript, we used the commercially available assays for phosphatidylinositol-4,5-bisphosphate 3-kinase catalytic subunit-α (PIK3CA), PIK3C3, and PIK3CD. PIK3C2B, PIK3CG, and PIK3C2G fusion vectors were obtained from Promega’s Tailored R&D Solutions group; tracers were developed in-house.

### C2C12 cells

C2C12 cells are a mouse myoblast cell line (CRL-1772, ATCC) cultured in DMEM with 20% FBS. *MTM1*-null clones were generated by genOway through targeting of exon 5 with either insertion of a stop or indel sequence. WT or the resulting *MTM1*-null C2C12 cells were then transduced with either nontargeting shRNA (SCH016H, MilliporeSigma) or PIK3C2B targeting shRNA (TRNC0000360889, CTTCATCATGGTGATGCATAT, MilliporeSigma) at an MOI of 1:10. Cells were then selected with 5 μg/mL puromycin (J67236.XF, Thermo Fisher Scientific) for 14 days and subsequently maintained at 2 μg/mL puromycin until time of assay.

### C2C12 cell imaging assay

First, 5,000 cells/well were plated in a 96-well PhenoPlate (Revvity). Cells were allowed to adhere and grow for 48 hours before growth media was replaced with differentiation media, DMEM with 2% horse serum (Invitrogen). After 72 hours, media was replenished and compounds were added at the indicated concentrations. Cells were treated with compounds for 48 hours before a 15-minute fixation by addition of 4% paraformaldehyde in PBS with 4% sucrose to media. Cells were washed twice with Tris-buffered saline (TBS) and then permeabilized for 5 minutes with 0.1% TBS Triton X-100. After a TBS wash, cells were incubated with 10% neutral goat serum in TBS overnight at 4°C, followed by an overnight incubation with primary anti-myosin antibody (MAB4470, MF20, R&D Systems) at a 1:1,000 dilution. Cells were then washed 3 times with 0.05% TBS Tween-20 and incubated (1:1,000) with Alexa Fluor 568 secondary antibody (A-21144, Invitrogen) for 2 hours, after which they were counterstained with 1 μg/mL DAPI (Invitrogen) for 30 minutes. Cells were then washed 3 times with 0.05% TBS Tween-20, covered with 50% glycerol, and covered with aluminum plate covers (Nunc).

Plates were analyzed using an Opera Phenix Plus High-Content imager (Revvity); all images were taken using the 20× confocal setting. The number of nuclei within Alexa Fluor 568–positive objects was then quantified in addition to total positive area and number of individual objects.

### C2C12 cell mass spectrometry

C2C12 cells were cultured in DMEM with 20% FBS, harvested with 0.25% trypsin, and snap-frozen as pellets containing 3 million cells. Cell pellets were resuspended in lysis buffer from the PreOmics iST-BCT kit and heated at 90°C with shaking at 750 rpm for 10 minutes using an Eppendorf ThermoMixer C to achieve cell lysis. Lysates were subsequently sonicated for 10 minutes (30 seconds on/30 seconds off) using a QSonica 700 Microplate Sonicator to shear DNA. For each sample, 100 μg of protein was transferred to a 96-well Eppendorf Protein LoBind PCR plate and adjusted to a final volume of 50 μL with BCT lysis buffer. Samples were digested overnight with trypsin at 37°C with shaking at 750 rpm on an Eppendorf ThermoMixer C. Peptide cleanup was performed using PreOmics BCT Phoenix cartridges according to the manufacturer’s protocol. Eluted peptides were quantified for total protein, dried in a Savant SPD2030 SpeedVac (Thermo Fisher Scientific), and resuspended in 0.1% formic acid (MilliporeSigma). For LC-MS/MS analysis, 600 ng of peptides per sample were injected onto an Evosep One system coupled to a timsTOF-HT mass spectrometer (Bruker), using a 40 SPD Whisper Zoom method and diaPASEF acquisition. Raw data were processed using DIA-NN (v1.8) in library-free, high-precision mode.

### Mutant mouse strains

A *D1212A PIK3C2B*–constitutive mutant mouse model was generated at GenOway by inserting GAT->GCC within exon 25 to create a PIK3C2B kinase-dead knockin (KI) mouse in a C57BL/6 genetic background (Supplemental Figure 1A). Validated embryonic stem cell clones were injected into blastocysts to generate chimeric animals. Progeny were genotyped by PCR (forward: 5′-GCAAATCCAGACAGGAAGACCTTAGTGG-3′ and reverse: 5′-CAGGAAGTGTGAAGCTGCCTCAGCT-3′) to detect either a 254 bp WT allele or a 345 bp KI allele. A subset of PCR-validated animals underwent further analysis by DNA sequencing to fully verify the integrity of the targeted region.

The *MTM1*-null mouse in a *129S5 × C57BL/6J-Tyr^c-Brd^* background was acquired from Taconic (model TF0892). The following genotyping primers were used to detect either the 280 bp WT allele or 241 bp KO allele: forward 5′-TCTCAGTGTTTAGGATTGAATCAGG-3′, WT reverse 5′-CCCAGGTGGTTCTAATGAGC-3′, and KO reverse 5′-ATAAACCCTCTTGCAGTTGCATC-3′.

The *MTM1* conditional KO mouse was generated at Mirimus by introducing loxP sequences upstream and downstream of exon 4 in pure C57BL/6N ES cells using CRISPR/Cas9 technology (Supplemental Figure 2A). Progeny were genotyped using PCR (forward 5′-TTGTCAACCTACCACCCAGC-3′ and reverse 5′-GTAGACCAGAGCCAGAGCAC-3′) to detect either the 155 bp WT allele or the 195 bp KI allele. A subset of PCR-validated animals underwent further analysis by DNA sequencing to fully verify the integrity of the targeted region. *Rosa26-CreER^T2^* transgenic mice (B6.129-Gt(ROSA)26Sortm1(cre/ERT2)Tyj/J) (57) were acquired from The Jackson Laboratory (stock 008463).

### Housing and husbandry

Mice included in this study were housed either at Charles River Laboratories or at Blueprint Medicine Corporation’s animal care facility. Mice were housed in temperature- and humidity-controlled rooms, with a 12-hour light/12-hour dark cycle with 10–15 room air changes per hour. Animals were housed in disposable, individually ventilated rodent cages (Innovive) prefilled with Alpha-Dri bedding (Innovive) and a minimum of 2 enrichment devices. Animals not breeding were fed irradiated PicoLab 5053 pelleted rodent chow (LabDiet) and allowed access to acidified water, ad libitum. Breeding animals were fed irradiated PicoLab 5058 chow (LabDiet), consisting of 20% protein and 9% fat pelleted diet, and allowed access to acidified water, ad libitum.

### Oral dosing of PIK3C2B inhibitors

Animals in the study were weaned on P21 and given a combination of electrolyte gel (Bio-Serve), transgenic dough diet (Bio-Serv), and a small amount of irradiated PicoLab 5058 pelleted diet (LabDiet) containing 20% protein and 9% fat. Animals were enrolled in the study based on their date of birth. Weights were measured at the beginning of the study and before each dose administration. Formulations of BLU3797 were prepared immediately prior to dose administration. A solution of 0.5% (w/v) of sodium carboxymethyl cellulose and 1% (v/v) Tween 80 in water was used as vehicle. Any animal found to be in a moribund state was removed from the study. Animal dosing was initiated on P22 unless otherwise stated.

Terminal whole blood samples were collected via cardiac puncture. Muscle tissue (gastrocnemius and tibialis anterior) was snap-frozen or harvested and fixed in 10% neutral buffered formalin for 48 hours before being placed in 70% ethanol prior to processing for histology.

### In vivo phenotypic studies

The wire hanging test recommended by the TREAT-NMD Neuromuscular Network for evaluating overall subacute muscle function and coordination in mice (SOP DMD_M.2.1.004) was utilized in this study. Motor coordination, grip strength, and muscle function were assessed by recording how long a mouse could hold onto an inverted wire cage top, up to a maximum of 60 seconds. Mice were subjected to the MotoRater test (TSE systems) at Charles River Laboratories for fine-motor-kinematic analysis. Mice were acclimatized to the testing room for a minimum of 30 minutes before their walking motion was recorded by high-speed cameras (300 FPS) positioned above, below, and on either side of the animal. Videos of each mouse were then processed using Simi Motion software, and a custom-made automated analysis system was employed to analyze various gait patterns and movements (58–60).

### Histological analysis

IHC staining of laminin in FFPE mouse gastrocnemius muscle was conducted on the Bond RX platform (Leica Biosystems) using standard chromogenic methods. Prior to staining, slides were incubated with Proteinase K for 5 minutes at room temperature followed by a 45-minute incubation with laminin antibody (ab11575, Abcam). Antibody binding was detected with HRP-conjugated anti-rabbit secondary polymer, and visualization was achieved through chromogenic staining with 3,3’-DAB. Nuclei were visualized using a hematoxylin counterstain. Sections were digitally scanned using an Aperio AT2 whole slide scanner (Leica Biosystems). Image analysis of the digital slide images was performed using Oncotopix Discovery software (Visiopharm).

To determine the percentage of myocytes containing central nuclei, a manual counting method was employed. The entire muscle sample was thoroughly examined, and representative fields of view were selected based on notable myofiber changes. In each of the 5 high-powered fields (400×), 30 myocytes were counted to obtain accurate data.

To enhance the identification of myocytes, a deep-learning artificial intelligence (AI) algorithm was trained. The IHC-stained slide was utilized to define a specific region of interest (ROI) that encompassed the cross-sectional muscle tissue. The AI algorithm processed these ROIs to accurately identify myocytes. The diameter of each identified myocyte was measured along its lesser diameter, providing additional quantitative information.

### RNA analysis

Total RNA was obtained from tissues using the miRNeasy Tissue/Cells Advanced Micro kit (217684, QIAGEN) following the manufacturer’s instructions. Isolated miRNAs were analyzed using the NanoString V1.5 miRNA kit (150649, NanoString) and nCounter following the manufacturer’s instructions. Relative miRNA levels and group comparisons were made using ROSALIND (Rosalind.bio). qPCR analysis of miRNAs was performed using miRCURY LNA miRNA PCR assay kits (QIAGEN). *Smad1*, *Sfrp1*, and *Gapdh* mRNA abundance was assessed using TaqMan Probes (Thermo Fisher Scientific) from cDNA generated using Superscript IV (Thermo Fisher Scientific).

### Detection of muscle PtdIns3P, SQSTM1/P62, SFRP1, and plasma myostatin

PtdIns3P was extracted from muscle using a lipid extraction kit (Abcam). Briefly, gastrocnemius muscles were collected and snap-frozen in liquid nitrogen before being moved to a 2 mL microcentrifuge tube containing metal beads (Cayman Chemical). Next, 500 μL of lipid extraction buffer was added to the sample, mixed at room temperature for 15 minutes, and homogenized in a Precellys 24 Touch instrument (Cayman Chemical). Lysate was transferred to a clean microcentrifuge tube and spun at 10,000*g* for 5 minutes. The supernatant was collected and dried using a vacuum drier. PtdIns3P was detected using a PtdIns3P mass ELISA (Echelon Biosciences) according to the manufacturer’s instructions.

To assess SQSTM1/P62 or SFRP1 levels, muscles were homogenized in RIPA buffer (889900, Thermo Fisher Scientific), supplemented with cOmplete EDTA-free protease inhibitor cocktail, 50 mM sodium fluoride, 1 mM phenylmethanesulfonyl fluoride, 1 mM sodium orthovanadate, 2 mM β-glycerophosphate (all from MilliporeSigma), and HALT protease and phosphatase inhibitor cocktail (Thermo Fisher Scientific). For SQSTM1/P62, homogenates were clarified and 5–20 μg of total protein was diluted in assay buffer and analyzed using a SQSTM1/P62 ELISA kit (Enzo Life Sciences) following the manufacturer’s instructions. SFRP1 was assessed in a similar fashion using the SFRP1 ELISA kit (Antibodies.com). Myostatin levels were quantified from mouse plasma samples using the Quantikine Myostatin/GDF-8 ELISA kit (R&D Systems) following the manufacturer’s instructions.

### Western blotting

Muscles were processed as above but denatured in loading buffer (Bio-Rad Laboratories), run on TGX gels, and transferred using semi-dry transfer packs (Bio-Rad Laboratories). Cells were harvested on ice using PhosphoSafe extraction buffer (MilliporeSigma) with HALT protease-phosphatase inhibitors (Thermo Fisher Scientific). Membranes were blocked and stained using LI-COR TBS blocking buffer and visualized using a LI-COR Odyssey. Secondary antibodies were goat anti-mouse 680 (926-68070, LI-COR) and goat anti-rabbit 800 (926-32211, LI-COR) used at 1:5,000. Primary antibodies were mouse-anti GAPDH (5174, Cell Signaling Technology), rabbit-anti SQSTM1/P62 (5114, Cell Signaling Technology), rabbit-anti SMAD1 (6944, Cell Signaling Technology), mouse anti-myosin heavy chain (MAB4470, MF20, R&D Systems), mouse anti-myogenin (15-5643-82, Thermo Fisher Scientific), mouse anti-MyoD (MA1-41017, Thermo Fisher Scientific), mouse anti-PAX7 (MAB1675, R&D Systems), rabbit anti-actin (8456, Cell Signaling Technology), mouse anti-PIK3C2B (24788-1-AP, Proteintech), and rabbit anti-MTM1 (PA5-17972, Thermo Fisher Scientific), all used at 1:1,000 dilutions. Mouse anti–β-actin (3700, Cell Signaling Technology) was used at a 1:5,000 dilution.

### Proteomics/phosphoproteomics and lipidomics analysis

Analysis of tandem mass tag–labeled peptides to capture the proteome and phosphoproteome in *PIK3C2B^+/+^* *MTM1^–/y^* and *PIK3C2B^D1212A/D1212A^* *MTM1^–/y^* mouse muscles was performed using LC-MS/MS at IQ Proteomics. For both readouts, UniProt Mouse (2018) was used for peptide assignment, and a target-decoy strategy through weighted linear discriminant analysis was used to filter out false-positive peptides. Differential abundance was evaluated using empirical Bayes moderated 2-tailed *t* tests (61) and adjusted for FDR. Protein-protein interaction and pathway analysis of differentially abundant genes was performed using the STRING database (62). Quantification of lipid molecules in *PIK3C3^–/–^* or *PIK3C3^+/+^* C2C12 cells treated with a PIK3C2B inhibitor or with DMSO was performed at Lipotype GmbH with their Shotgun Lipidomics platform. Briefly, Orbitrap mass spectrometry with lipid class–specific internal standards provided absolute quantification of lipids. A proprietary software program, LipotypeXplorer, using molecular fragmentation query language identified lipids in the mass spectra, and further data processing and analysis were performed using the Lipotype library information and management system. Lipids were assigned to lipid classes for further analysis. Data were log-transformed and *z* scores scaled for differential abundance analysis using empirical Bayes moderated 2-tailed *t* tests (61).

### Statistics

Unless stated otherwise, descriptive statistics were performed with either means, quartiles, and ranges (box-and-whisker plots) or mean ± SEM. Comparisons of myocyte fusion, number, and nuclei fraction in C2C12 cells were done with 1-way ANOVA with Dunnett’s correction for multiple comparisons. Comparisons of wire hanging latency, percentage of myofibers with central nuclei, myofiber diameter (in *MTM^–/y^*/BLU3797 experiments), plasma myostatin, and myofiber nuclei density were done with Kruskal-Wallis test with Dunn’s correction for multiple comparisons. Comparisons of kinematic gait analyses, muscle SQSTM1/P62 expression (across BLU3797 dose levels), plasma myostatin levels, and SFRP1 expression in KO versus WT C2C12 cells were done with 1-way ANOVA with Tukey’s correction for multiple comparisons. Comparisons of myofiber diameter (in *MTM^–/y^*/PIK3C2B kinase-dead experiments) and muscle PtdIns3P expression were done with 1-way ANOVA with Holm-Šídák’s correction for multiple comparisons. Comparisons of select miRNAs across BLU3797 dosing regiments were made with an unpaired 2-tailed *t* test. Comparisons of muscle SQSTM1/P62 expression between *MTM1* WT and *MTM1*-null at varying BLU3797 treatment initiation timings were done with 1-way ANOVA with Šídák’s correction for multiple comparisons. *P* values less than 0.05 were considered significant.

### Study approval

No studies involved human participants. All animal experiments were conducted in full compliance with local, national, ethical, and regulatory principles and local licensing regulations under Blueprint Medicines Corporation IACUC–approved protocols following US Department of Agriculture guidelines and regulations for research.

### Data availability

The underlying data for the graphs are available in the Supporting Data Values file included as part of the supplemental material to this article.

## Author contributions

JCL, SS, and KF contributed to the original research ideas and evolution of overarching research goals and aims. JCL, AS, MLB, and JH contributed to the development of methodology and creation of models. MLB, AS, and MMC contributed to the verification, whether as a part of the activity or separate, of the overall replication, reproducibility of results, experiments, or other research outputs. MLB, AS, and MMC contributed to the application of statistical, mathematical, computational, or other formal techniques to analyze or synthesize study data. MLB, TS, EP, AS, JH, and GK contributed to the conduct of research and investigational processes, specifically performing the experiments or collecting data and evidence. MLB, TS, MMC, EP, JB, and AS contributed to the provision of study materials, reagents, laboratory samples, animals, instrumentation, computing resources, or other analysis tools. MLB, AS, and MMC contributed to management activities to annotate (produce metadata), scrub data, and maintain research data for initial use and later reuse. JCL and AS contributed to the preparation, creation, or presentation of the published work, specifically writing the initial draft. MLB, JCL, TS, AS, MMC, JH, GK, EP, JB, KF, and SS contributed to the preparation, creation, or presentation of the published work by those from the original research group, specifically critical review, commentary, or revision — including at pre- or post-publication stages. MLB, JCL, AS, and JH contributed to the preparation, creation, or presentation of the published work, specifically visualization and data presentation. JCL, TS, SS, and KF contributed to the oversight and leadership responsibility for the research activity planning and execution, including mentorship external to the core team. MLB, TS, JCL, and AS contributed to the management and coordination responsibility for the research activity planning and execution.

## Conflict of interest

AS, MLB, MC, TS, JH, GK, EP, JCL, and JB are current employees of Blueprint Medicines Corporation, a wholly owned subsidiary of Sanofi. KF and SS are former employees of Blueprint Medicines Corporation, a wholly owned subsidiary of Sanofi.

## Funding support

- Blueprint Medicines Corporation, a wholly owned subsidiary of Sanofi.

## Supplementary Material

Supplemental data

Unedited blot and gel images

Supporting data values

## Figures and Tables

**Table 1:**
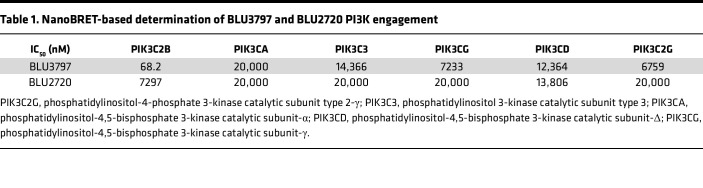
NanoBRET-based determination of BLU3797 and BLU2720 PI3K engagement

## Version 1 04/09/2026

In-Press Preview

## Version 2 05/22/2026

Electronic publication
