# Supplementary material for: Pharmacological PIK3C2B inhibition rescues XLMTM phenotype in mouse models and identifies molecular markers of disease
Source: JCI Insight. 2026 Apr 9;11(10):e198568. doi: 10.1172/jci.insight.198568 (PMC13232722; doi:10.1172/jci.insight.198568)
Supplement: Supplemental data [file jciinsight-11-198568-s125.pdf]

Supplementary Material for

Pharmacological PIK3C2B inhibition rescues XLMTM phenotype in mouse models and identifies molecular markers of disease

Andrew Shearer[1], Melissa L. Brooks[1], Maxine M. Chen[1], Thiwanka Samarakoon[1], John Hsieh[1], Gramoz Kondakci[1], Emanuele Perola[1], Jason Brubaker[1], Kristina Fetalvero[1], Stefanie Schalm[1,2], Joana Caetano-Lopes[1,*]

[1]Blueprint Medicines Corporation, Cambridge, MA, USA. [2]Bayer Research & Innovation Center, Cambridge, MA, USA.

*Corresponding author

Supplemental figures

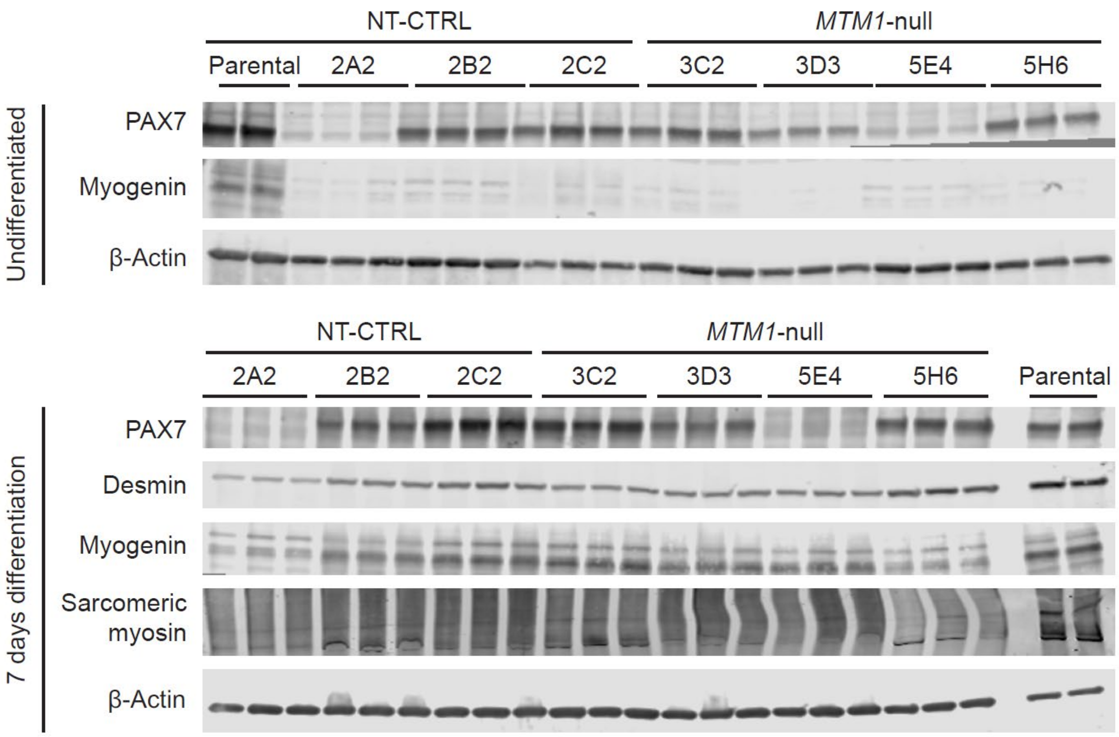

Supplemental Figure 1. Generation of C2C12 *MTM1*-null clones possess varied cellular phenotypes. Western blot analysis of clones that had been generated by CRISPR-Cas9 targeted ablation of *MTM1* or cells generated using a non-targeting guide.

KO, knock-out; MTM1, myotubularin 1; NT-CTRL, non-targeting control; sgRNA, single-guide RNA.

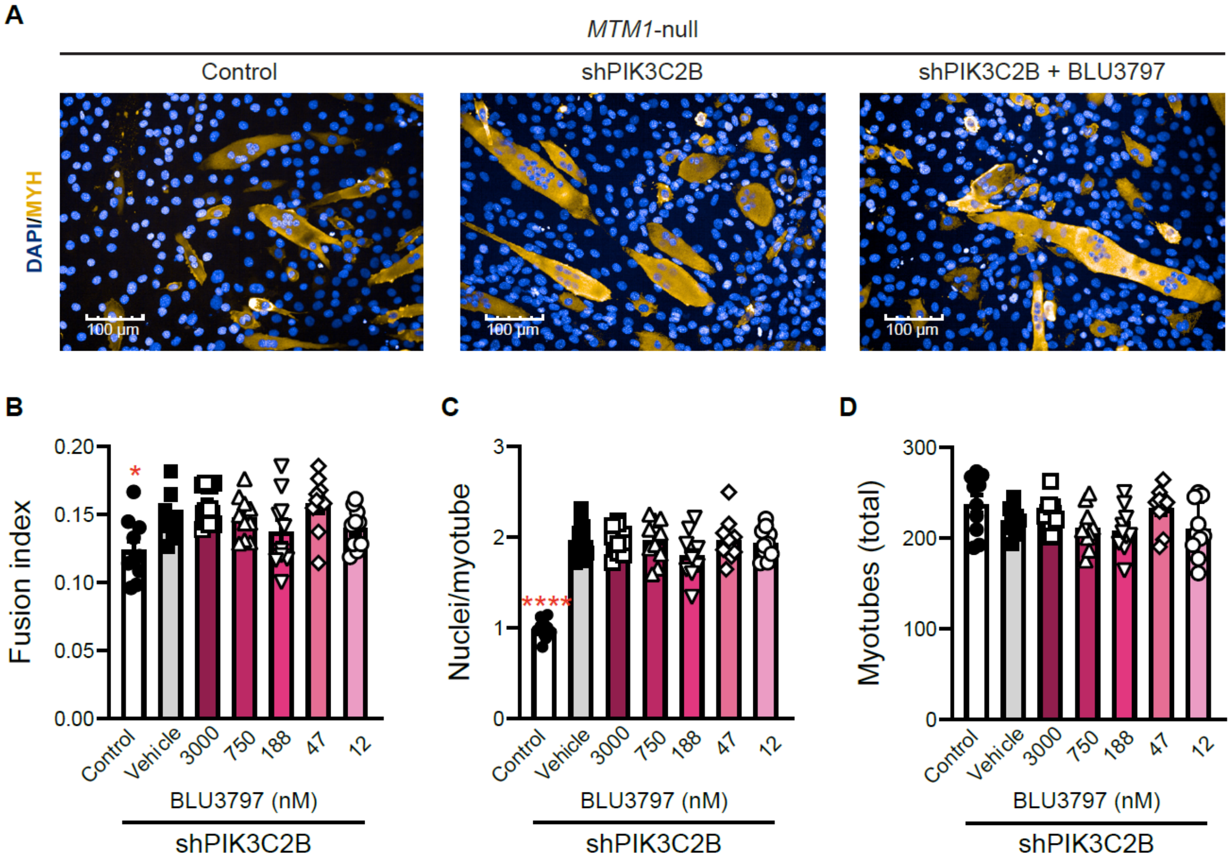

Supplemental Figure 2. PIK3C2B inhibition does not increase myocyte-myotube fusion in PIK3C2B-depleted *MTM1*-KO C2C12 cells. (A) Example images of *MTM1*$^{-/-}$ C2C12 cells fixed after 5 days differentiation and 2 days of BLU3797 treatment (experiment conducted twice). **(B)** Quantification of the fraction of nuclei in MYH positive structures (Fusion Index) from 5 random non-overlapping fields (n = 10). **(C)** Representative quantification of average nuclei in MYH positive structures (myotubes) from 5 random non-overlapping fields (n = 10). **(D)** Representative quantification of the total MYH positive structures (myotubes) from 5 random non-overlapping fields (n = 10). Error bars show SEM. *p≤0.05, **p≤0.01, ***p≤0.001, ****p≤0.0001. Comparison of myocyte fusion, number, and nuclei fraction in C2C12 cells were done by one-way analysis of variance (ANOVA) with Dunnett's correction for multiple comparisons.

DAPI, 4',6-diamidino 2-phenylindole; MTM1, myotubularin 1; MYH, myosin heavy chain; PIK3C2B, phosphatidylinositol-4-phosphate 3-kinase catalytic subunit type 2 beta; SEM, standard error of the mean.

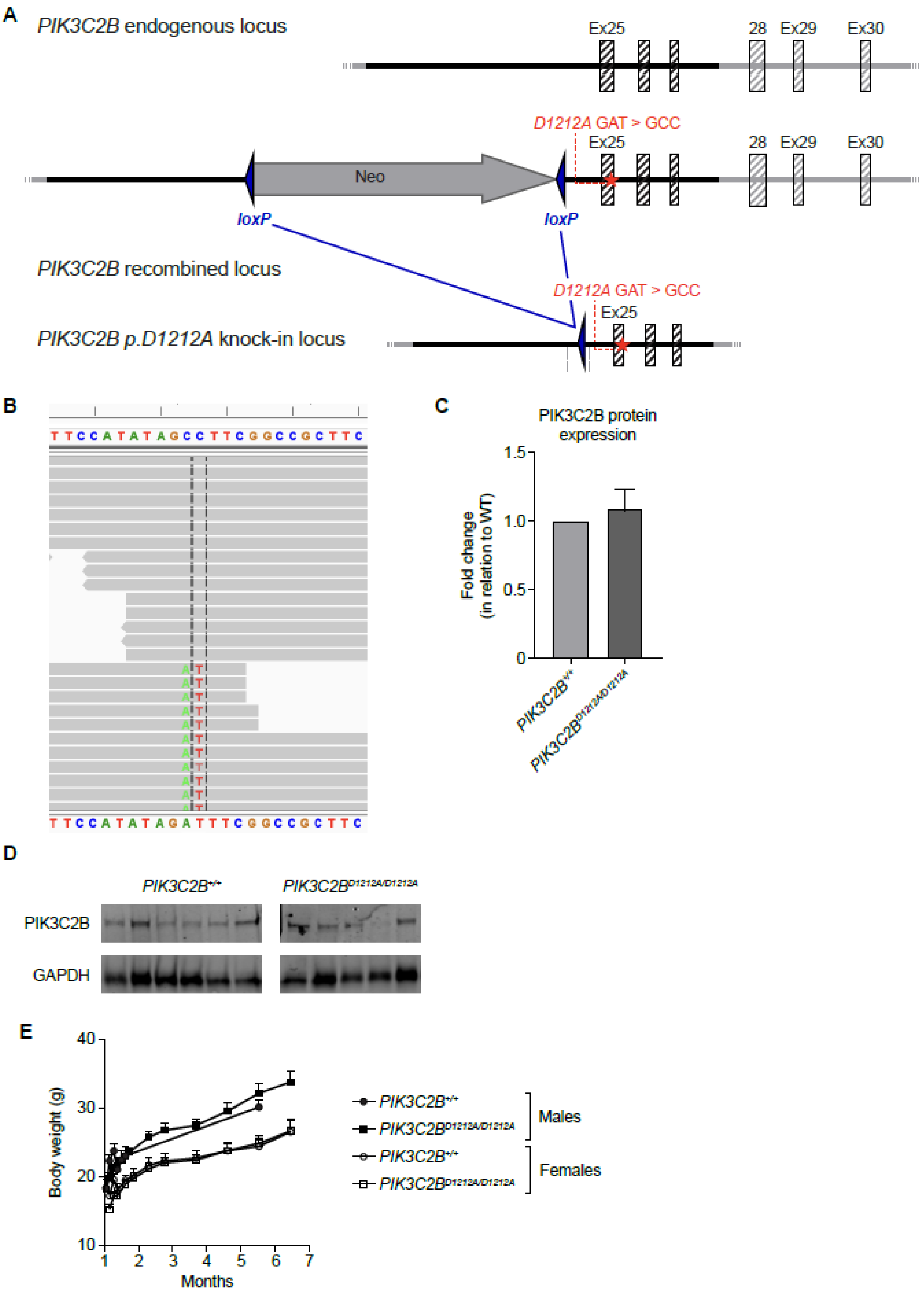
A
PIK3C2B endogenous locus
Ex25
28
Ex29
Ex30
D1212A GAT > GCC
Neo
loxP
loxP
PIK3C2B recombined locus
PIK3C2B p.D1212A knock-in locus
B
TTCCATATAGCCTTCGGCCGCTTC
TTCCATATAGATTTCGGCCGCTTC
C
PIK3C2B protein expression
Fold change (in relation to WT)
1.5
1.0
0.5
0
PIK3C2B+/+
PIK3C2BD1212A/D1212A
D
PIK3C2B+/+
PIK3C2BD1212A/D1212A
PIK3C2B
GAPDH
E
Body weight (g)
40
30
20
10
1
2
3
4
5
6
7
Months
PIK3C2B+/+
PIK3C2BD1212A/D1212A
Males
PIK3C2B+/+
PIK3C2BD1212A/D1212A
Females

32

Supplemental Figure 3. The *D1212A* mutation was introduced to generate a kinase dead mouse model of PIK3C2B. (**A**) Generation of the D1212A kinase dead mouse model by GAT→GCC insertion within exon 25 of C57BL/6 ES cells. A floxed Neomycin resistance cassette was utilized for efficient selection of ES cells. Breeding was established with C57BL/6N Cre-deleter mice. This breeding strategy resulted in the generation of heterozygous mice carrying the *D1212A* knock-in allele. (**B**) Two CC mutations introduced are shown above in gray for each read, while the corresponding WT sequence is shown in green and red and spelled out at the bottom. (**C**) Comparison of PIK3C2B protein levels from mouse muscle lysate. (**D**) Comparison of body weight between male mice with the *D1212A* mutation ($PIK3C2B^{D1212A/D1212A}$) and those without the mutation ($PIK3C2B^{+/+}$), as well as between female mice with the *D1212A* mutation ($PIK3C2B^{D1212A/D1212A}$) and those without the mutation ($PIK3C2B^{+/+}$). Error bars show SEM.
ES, embryonic stem; PIK3C2B, phosphatidylinositol-4-phosphate 3-kinase catalytic subunit type 2 beta; SEM, standard error of the mean; WT, wild-type.

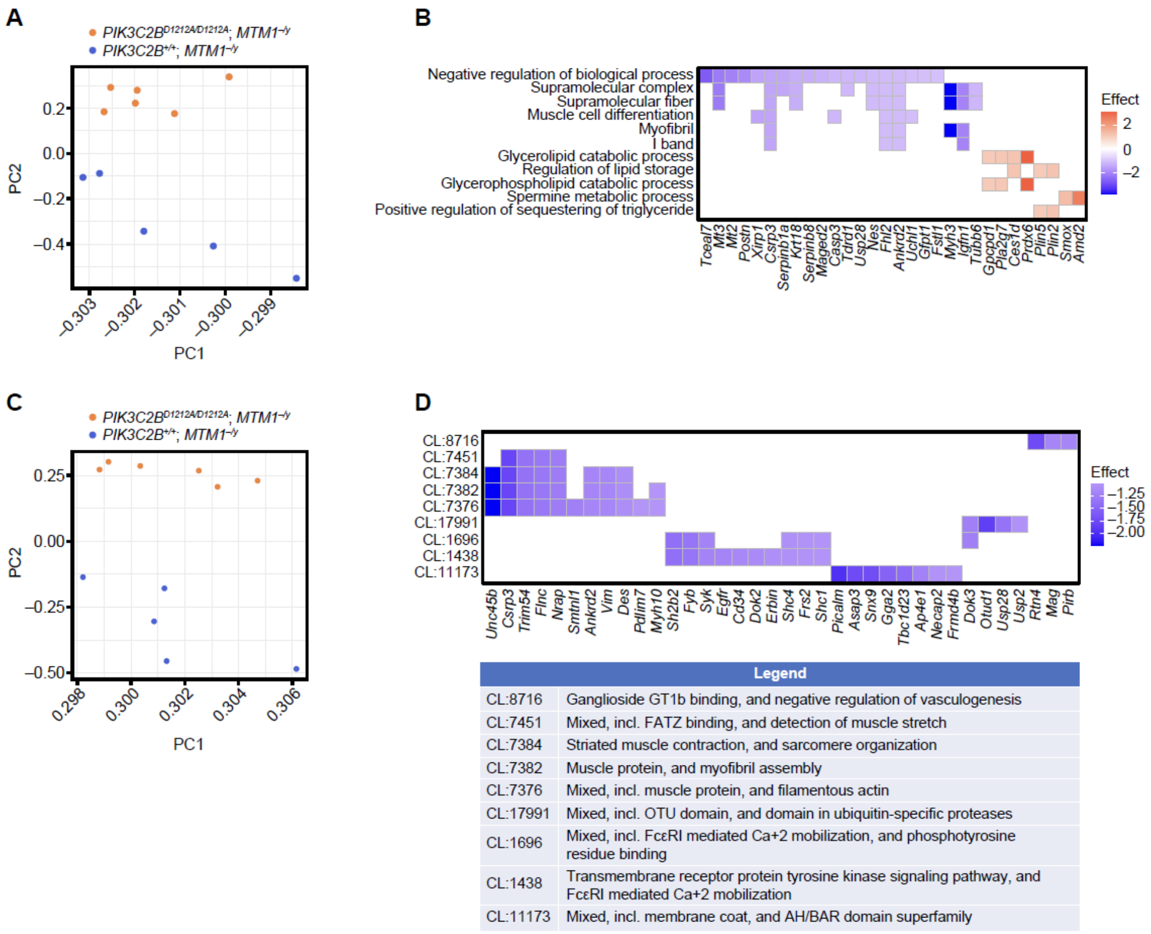

Supplemental Figure 4. Differential proteomics and phosphoproteomics abundance in PIK3C2B kinase dead *MTM1*$^{-/y}$ mice compared with *PIK3C2B*$^{+/+}$; *MTM1*$^{-/y}$ identifies genes involved in enrichment of muscle-related pathways. (**A**) Principal components analysis of protein abundance in *PIK3C2B*$^{D1212A/D1212A}$; *MTM1*$^{-/y}$ mice and *PIK3C2B*$^{+/+}$; *MTM1*$^{-/y}$ mice. (**B**). Heatmap of genes involved in the 10 GO most enriched pathways identified in differential abundance analysis of the proteome in *PIK3C2B*$^{D1212A/D1212A}$; *MTM1*$^{-/y}$ vs. *PIK3C2B*$^{+/+}$; *MTM1*$^{-/y}$ mice. (**C**) Principal components analysis of phosphoprotein abundance in *PIK3C2B*$^{D1212A/D1212A}$; *MTM1*$^{-/y}$ and *PIK3C2B*$^{+/+}$; *MTM1*$^{-/y}$ mice. (**D**) Heatmap of genes involved in the 10 most enriched GO pathways identified in differential abundance analysis of the phosphoproteome in *PIK3C2B*$^{D1212A/D1212A}$; *MTM1*$^{-/y}$ vs. *PIK3C2B*$^{+/+}$; *MTM1*$^{-/y}$ mice with *MTM1*$^{-/y}$. GO, gene ontology; MTM1, myotubularin 1; PIK3C2B, phosphatidylinositol-4-phosphate 3-kinase catalytic subunit type 2 beta.

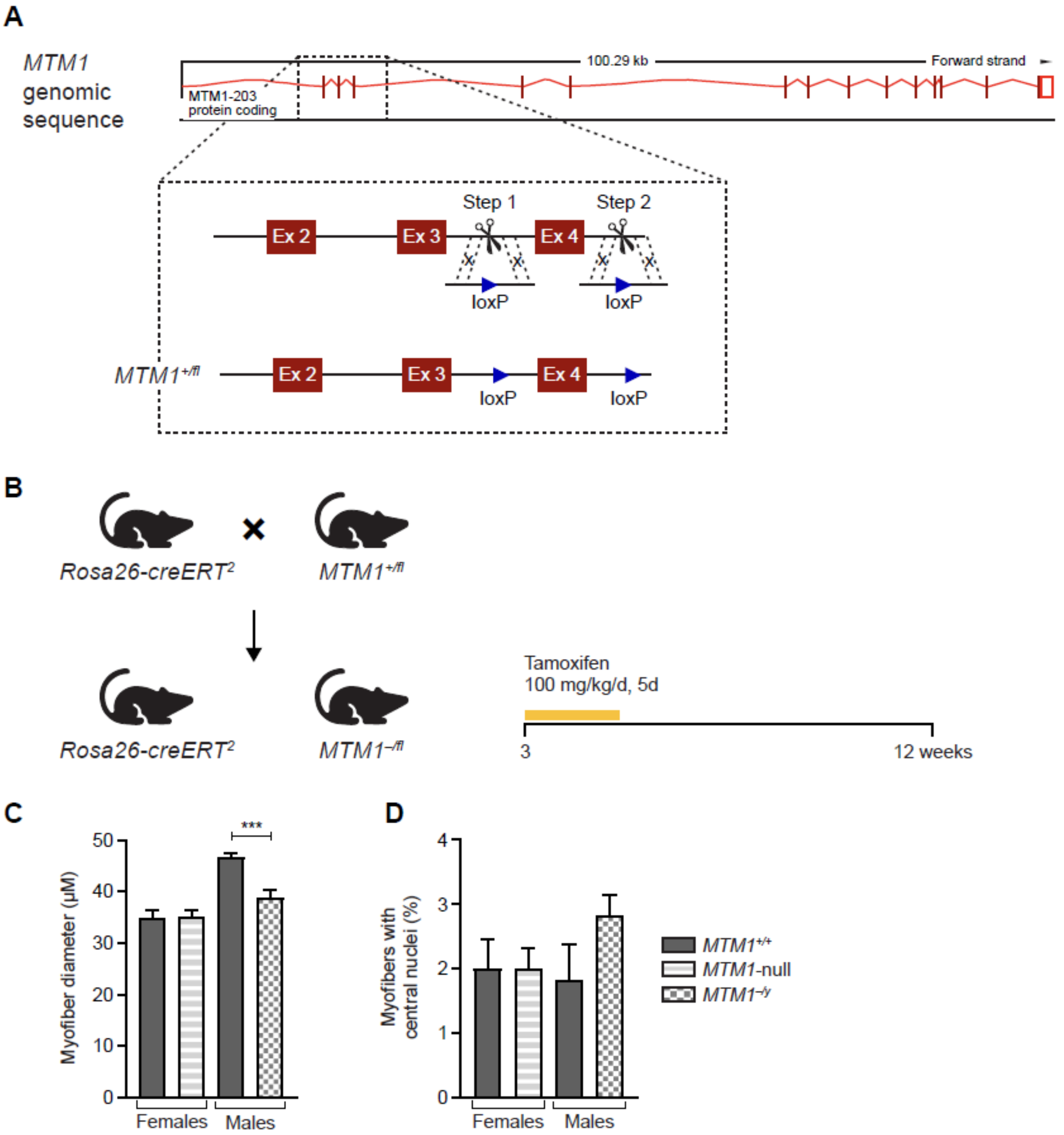

Supplemental Figure 5. The conditional knock-out of the *MTM1* gene in mice resulted in a subtle muscle phenotype specifically observed in male individuals. (**A**) Generation of the *MTM1*$^{+/fl}$ mouse, loxP sites were introduced upstream and downstream of *MTM1* exon 4 using CRISPR/Cas9. (**B**) Recombination of the floxed allele was induced by crossing *MTM1*$^{-/fl}$ animals with *Rosa26creER*^{T2} mice. Resulting offspring were treated with tamoxifen at a dose of 100 mg/kg/d for 5 consecutive days. (**C**) Summary of histological analysis of myofiber diameter in 12-week-old male and female mice. (**D**) Summary of histological analysis of the percentage of myofibers with central nuclei for both male and female muscle. Error bars show SEM. ***p≤0.001. Comparisons of myofiber diameter were done by Kruskal-Wallis test with Dunn’s correction for multiple comparisons.

MTM1, myotubularin 1; SEM, standard error of the mean.

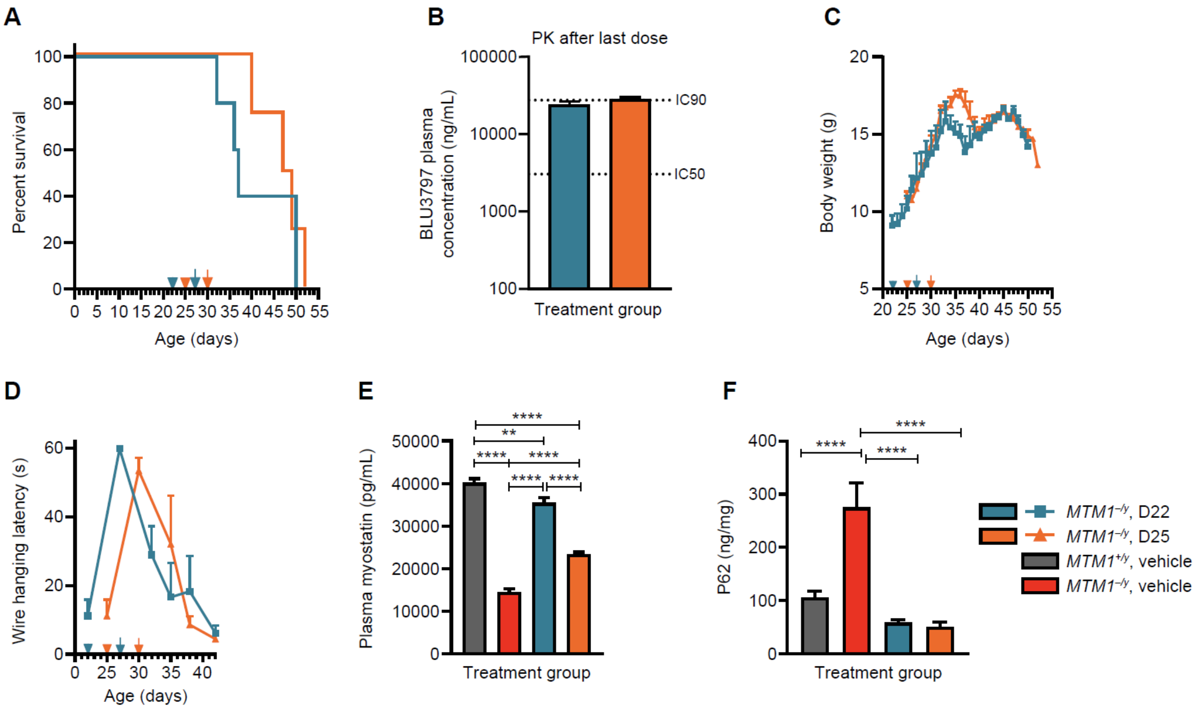

Supplemental Figure 6. BLU3797 effectively increases the lifespan of *MTM1$^{-/y}$* mice and restores muscle function, regardless of whether treatment is initiated on day 22 or day 25 of age. (**A**) Kaplan–Meier curve of *MTM1$^{-/y}$* animals that were given BLU3797 at a dose of 75 mg/kg, twice daily, starting either on day 22 (teal arrowhead) or day 25 (orange arrowhead) of age. Dosing stopped 5 days later (teal and orange arrows). (**B**) Plasma PK after the last dose of BLU3797. FBS-corrected NanoBRET™ IC_{50} and IC_{90} lines are indicated. (**C**) Animal weight. (**D**) Analysis of muscle function by hanging latency. (**E**) Measurement of plasma myostatin on the final dosing day, day 5 post first dose. (**F**) SQSTM1/P62 protein levels measured in the muscle at end of the study. Arrowheads represent start of the study; arrows represent the last day of dosing. Error bars show SEM. *$p < 0.05$; **$p < 0.01$; ***$p < 0.001$; ****$p < 0.0001$. Comparisons of plasma myostatin levels were done by one-way analysis of variance with Tukey's correction for multiple comparisons. Comparisons of muscle P62 expression were done by one-way analysis of variance with Šídák's correction for multiple comparisons.

FBS, fetal bovine serum; IC_{50}, half-maximal inhibitory concentration; IC_{90}, 90% inhibitory concentration; MTM1, myotubularin 1; PK, pharmacokinetics; SEM, standard error of the mean.

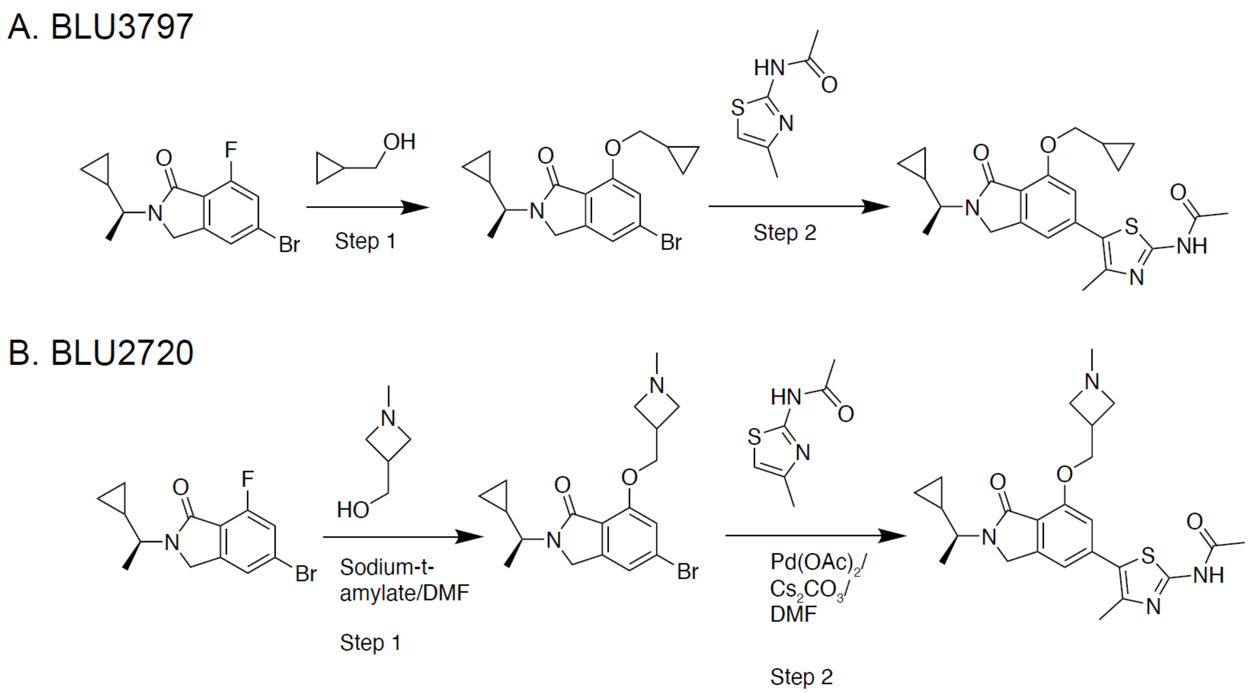

Supplemental Figure S7. Steps in the generation of BLU3797 and BLU2720. (**A**) Step 1 involves the synthesis of (S)-5-bromo-2-(1-cyclopropylethyl)-7-(cyclopropylmethoxy)isoindolin-1-one. Step 2 involves the synthesis of (S)-N-(5-(2-(1-cyclopropylethyl)-7-(cyclopropylmethoxy)-1-oxoisoindolin-5-yl)-4-methylthiazol-2-yl)acetamide. (**B**) Step 1 involves the synthesis of 5-bromo-2-[(1S)-1-cyclopropylethyl]-7-[(1-methylazetidin-3-yl)methoxy]isoindolin-1-one. Step 2 involves the synthesis of N-[5-[2-[(1S)-1-cyclopropylethyl]-7-[(1-methylazetidin-3-yl)methoxy]-1-oxo-isoindolin-5-yl]-4-methyl-thiazol-2-yl]acetamide.
DMF, N,N-Dimethylformamide.
