# Supplementary material for: Pharmacological PIK3C2B inhibition rescues XLMTM phenotype in mouse models and identifies molecular markers of disease
Source: JCI Insight. 2026 Apr 9;11(10):e198568. doi: 10.1172/jci.insight.198568 (PMC13232722; doi:10.1172/jci.insight.198568)

FIGURE 2 PANEL B

Figure 2B - PIK3C2B

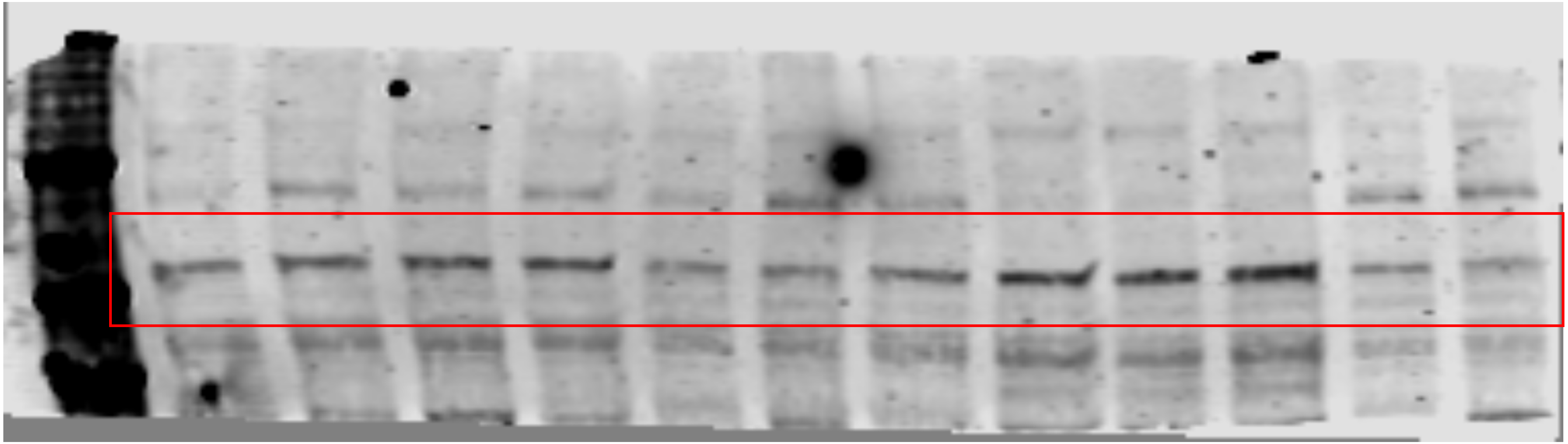

Figure 2B – PAX7

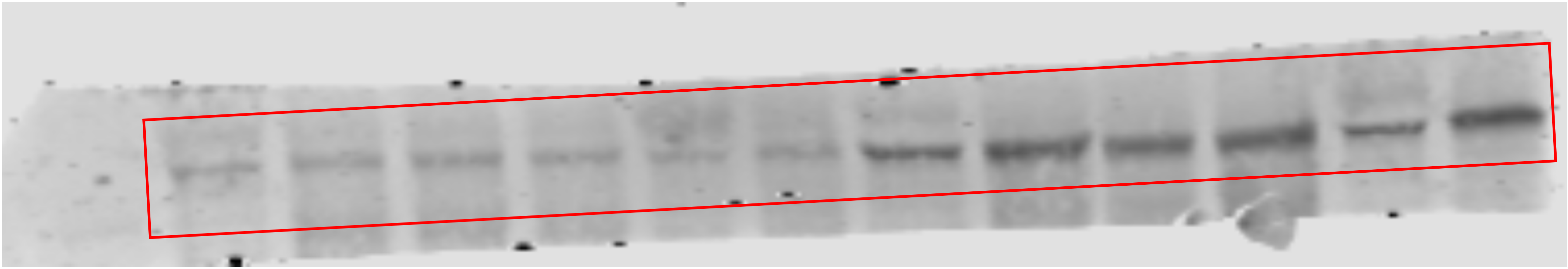

Figure 2B – MyoG

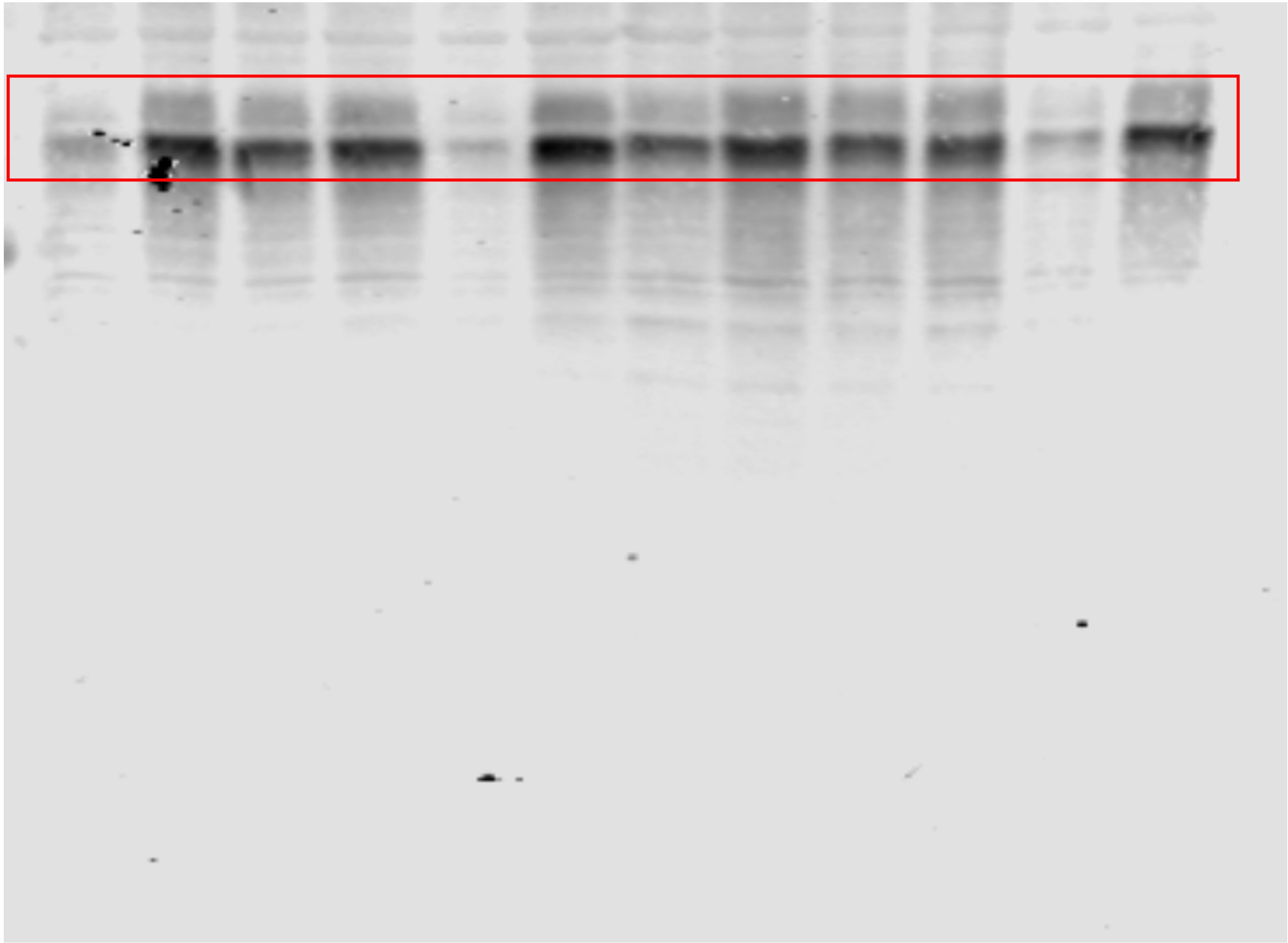

Figure 2B – MyoD

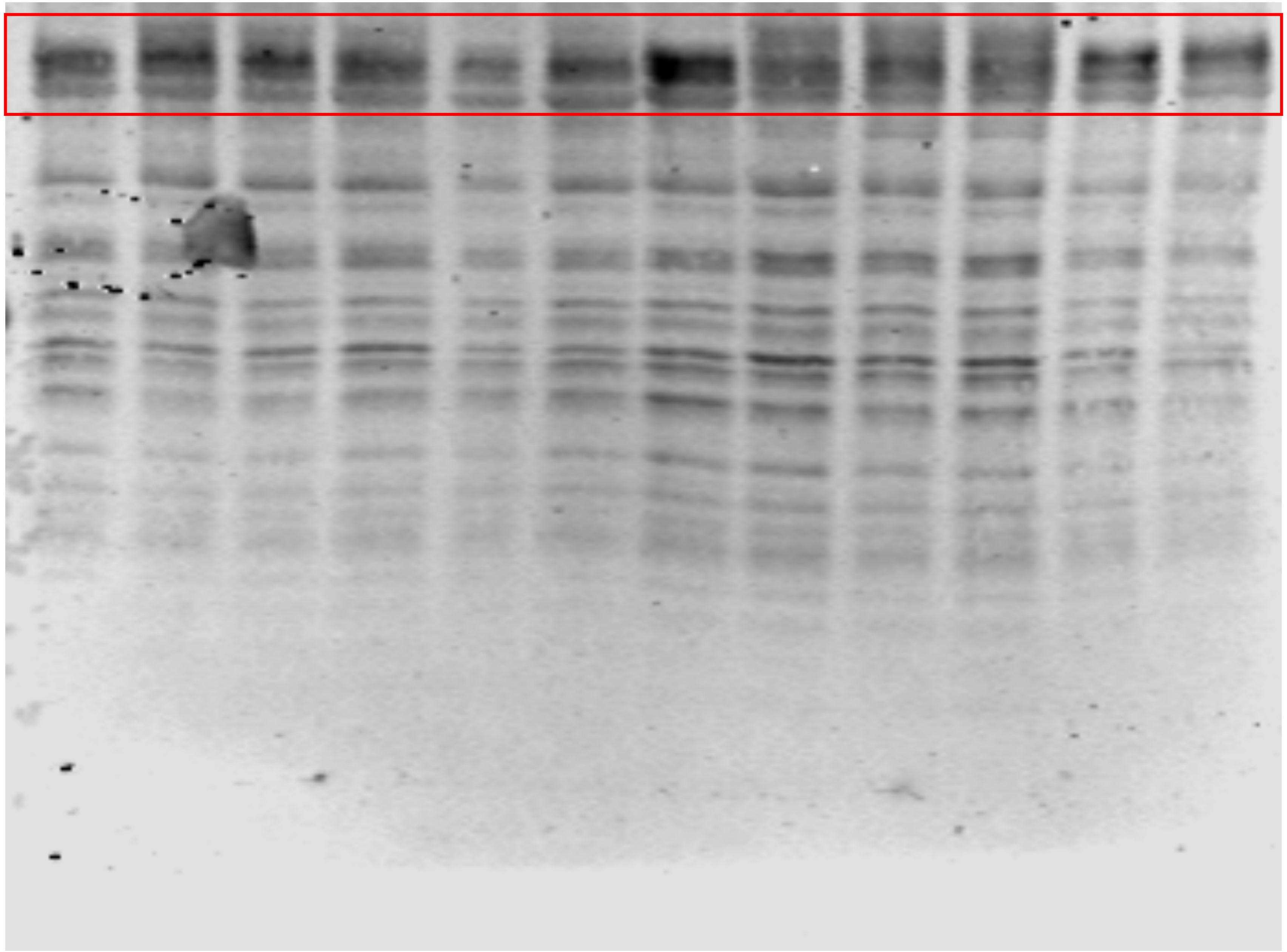

Figure 2B –β-actin

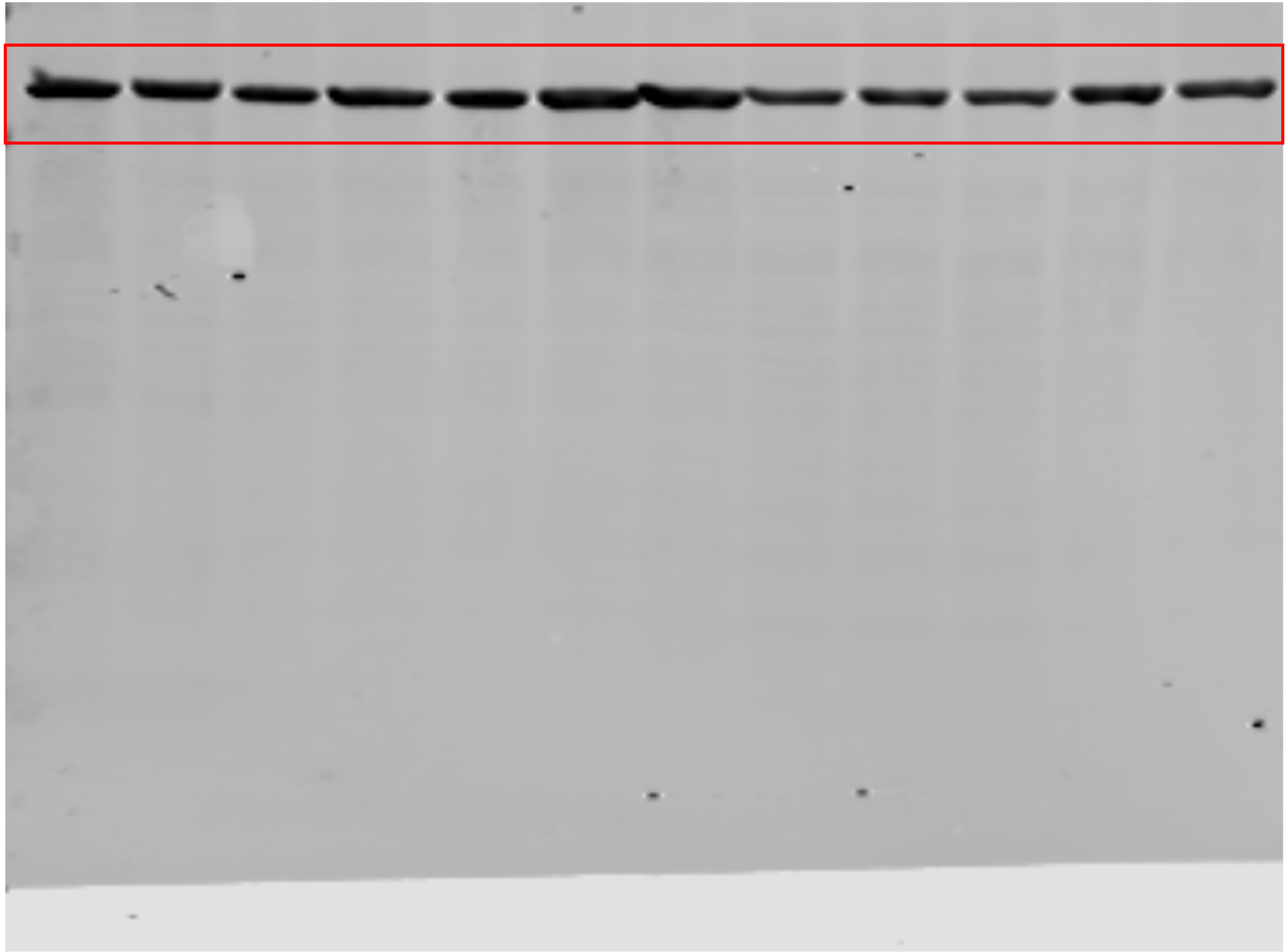

FIGURE 4 PANEL K

Figure 4K – SQSTM1/P62

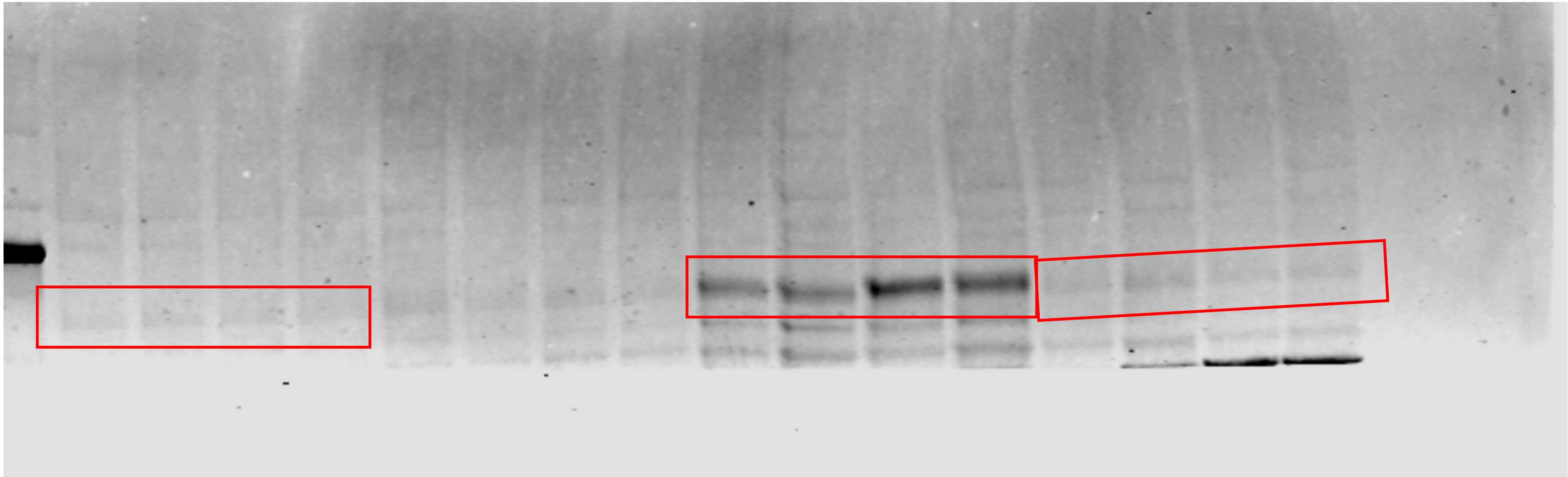

PIK3C2B +/+
MTM1 +/y

PIK3C2B +/+
MTM1 -/y

PIK3C2B D1212A/D1212A
MTM1 -/y

Figure 4K – GAPDH

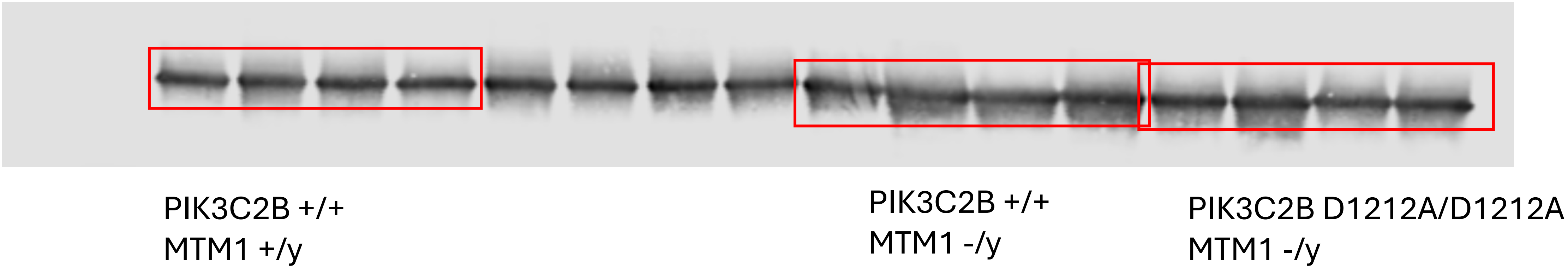

FIGURE 7 PANEL H

Figure 7H – SMAD1

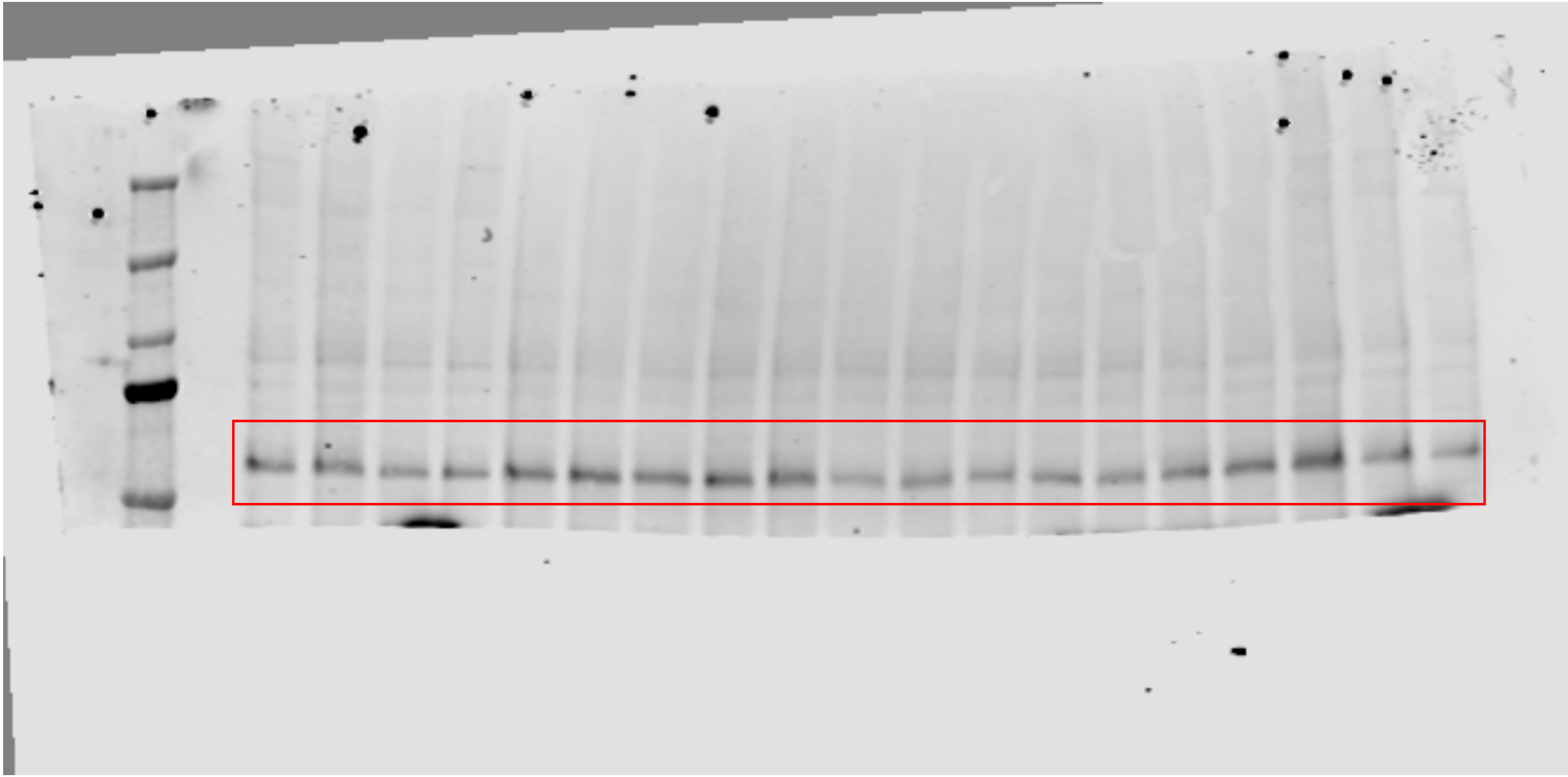

Figure 7H – Actin

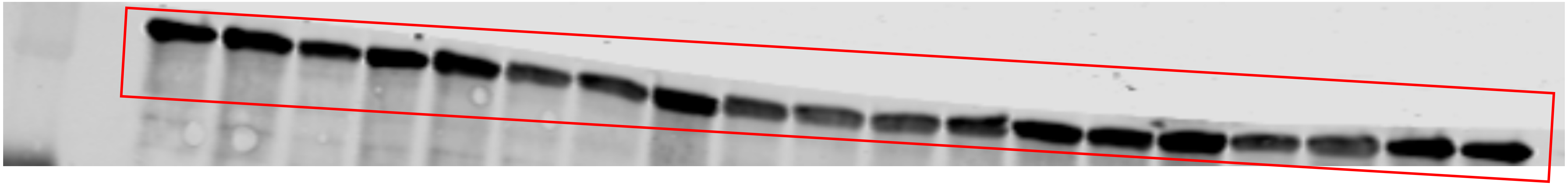

FIGURE S1

Suppl Figure S1 – Undifferentiated PAX7

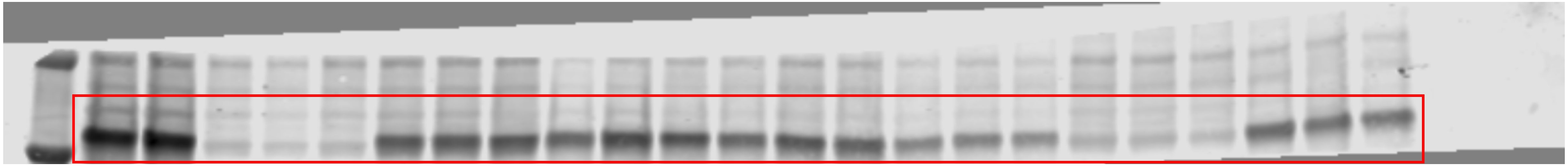

Suppl Figure S1 – Undifferentiated myogenin

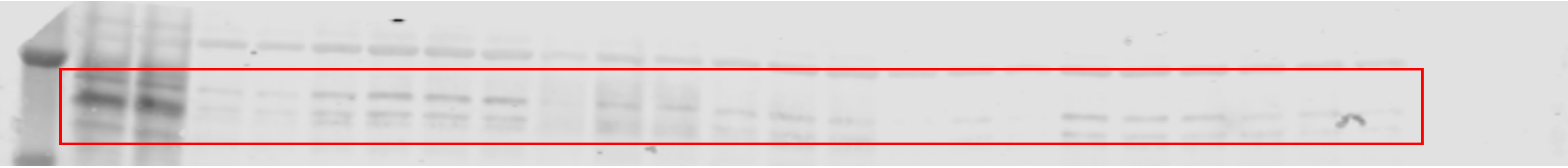

Suppl Figure S1 – Undifferentiated β-actin

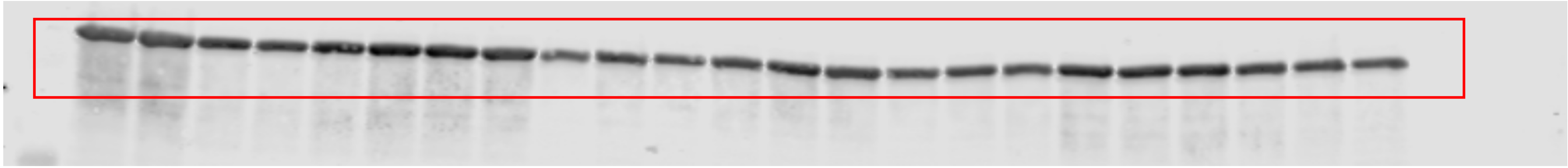

Suppl Figure S1 – Differentiated PAX7

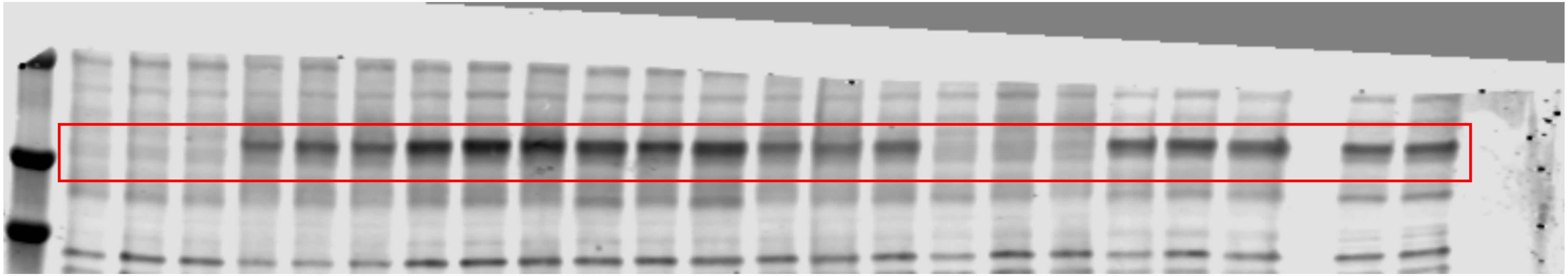

Suppl Figure S1 – Differentiated desmin

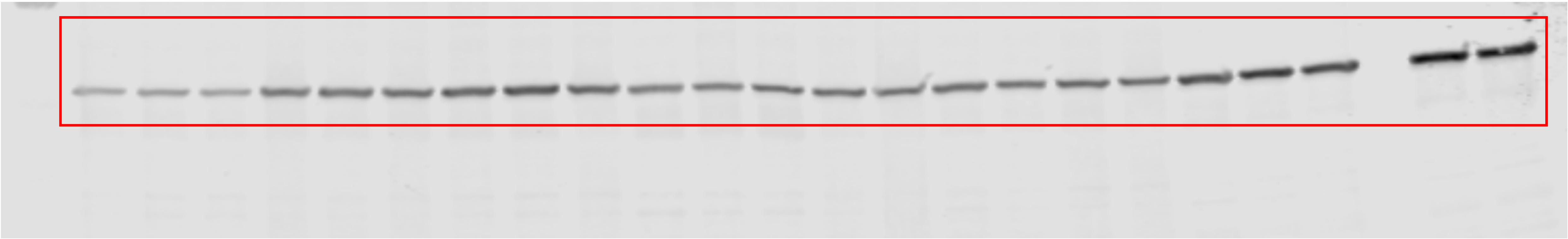

Suppl Figure S1 – Differentiated myogenin

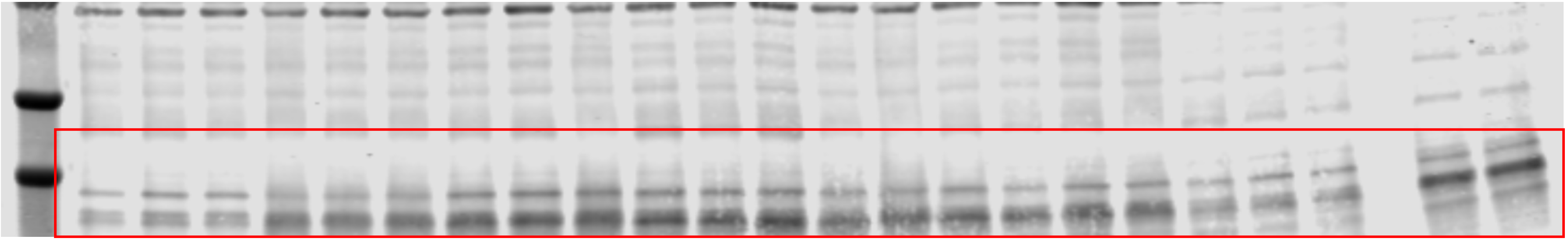

Suppl Figure S1 – Differentiated sarcomeric myosin

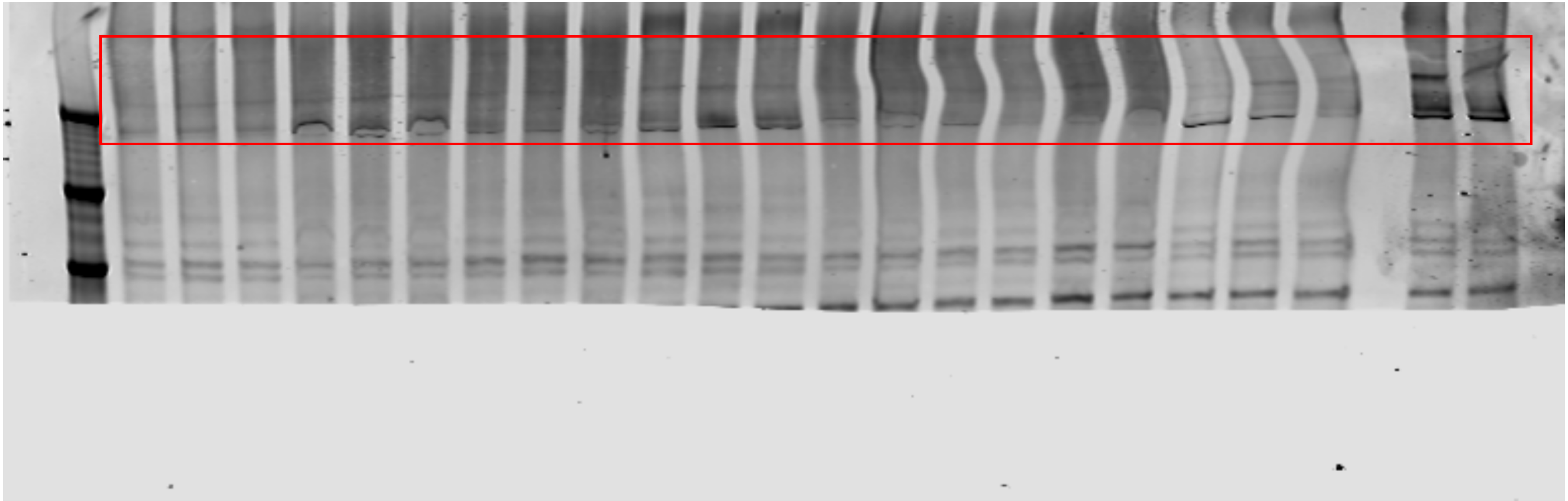

Suppl Figure S1 – Differentiated β-actin

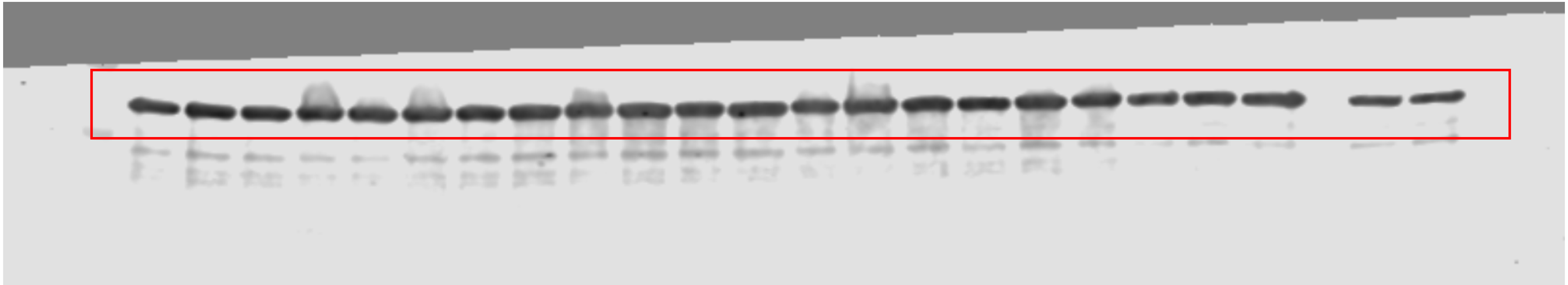

FIGURE S3

Suppl Figure S3 – GAPDH

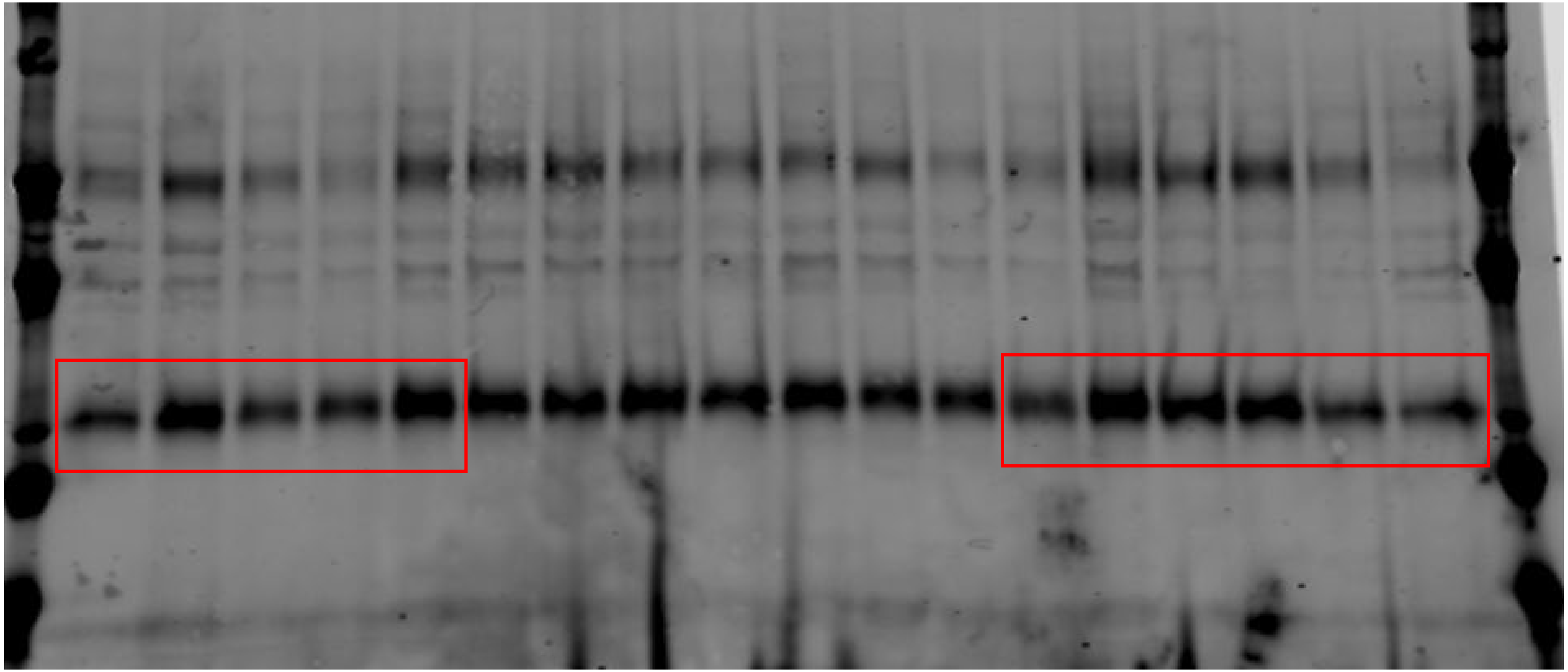

Suppl Figure S3 – PIK3C2B

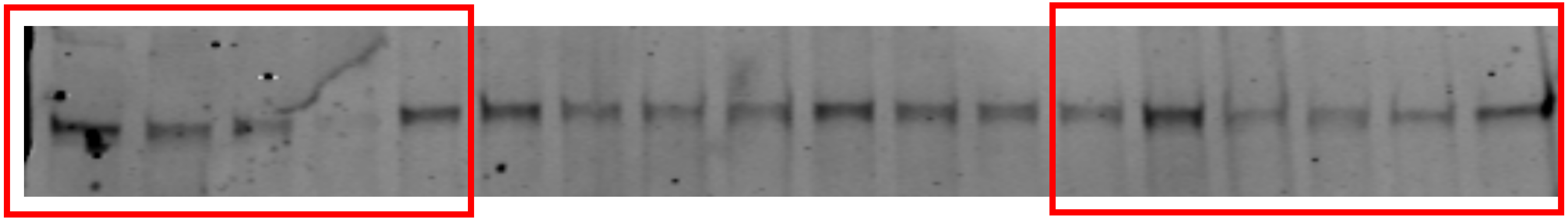

Supplement: Unedited blot and gel images [file jciinsight-11-198568-s126.pdf]
